# Switching of dominant retrotransposon silencing strategies from posttranscriptional to transcriptional mechanisms during male germ-cell development in mice

**DOI:** 10.1371/journal.pgen.1006926

**Published:** 2017-07-27

**Authors:** Kota Inoue, Kenji Ichiyanagi, Kei Fukuda, Michael Glinka, Hiroyuki Sasaki

**Affiliations:** 1 Division of Epigenomics and Development, Medical Institute of Bioregulation and Epigenome Network Research Center, Kyushu University, 3-1-1 Maidashi, Higashi-ku, Fukuoka, Japan; 2 Laboratory of Genome and Epigenome Dynamics, Department of Applied Molecular Biosciences, Graduate School of Bioagricultural Sciences, Nagoya University, Furo-cho, Chikusa-ku, Nagoya, Japan; 3 Cellular Memory Laboratory, RIKEN, Wako, Saitama, Japan; 4 Faculty of Science, University of Bristol, Bristol, United Kingdom; Cornell University, UNITED STATES

## Abstract

Mammalian genomes harbor millions of retrotransposon copies, some of which are transpositionally active. In mouse prospermatogonia, PIWI-interacting small RNAs (piRNAs) combat retrotransposon activity to maintain the genomic integrity. The piRNA system destroys retrotransposon-derived RNAs and guides *de novo* DNA methylation at some retrotransposon promoters. However, it remains unclear whether DNA methylation contributes to retrotransposon silencing in prospermatogonia. We have performed comprehensive studies of DNA methylation and polyA(+) RNAs (transcriptome) in developing male germ cells from *Pld6/Mitopld* and *Dnmt3l* knockout mice, which are defective in piRNA biogenesis and *de novo* DNA methylation, respectively. The *Dnmt3l* mutation greatly reduced DNA methylation levels at most retrotransposons, but its impact on their RNA abundance was limited in prospermatogonia. In *Pld6* mutant germ cells, although only a few retrotransposons exhibited reduced DNA methylation, many showed increased expression at the RNA level. More detailed analysis of RNA sequencing, nascent RNA quantification, profiling of cleaved RNA ends, and the results obtained from double knockout mice suggest that PLD6 works mainly at the posttranscriptional level. The increase in retrotransposon expression was larger in *Pld6* mutants than it was in *Dnmt3l* mutants, suggesting that RNA degradation by the piRNA system plays a more important role than does DNA methylation in prospermatogonia. However, DNA methylation had a long-term effect: hypomethylation caused by the *Pld6* or *Dnmt3l* mutation resulted in increased retrotransposon expression in meiotic spermatocytes. Thus, posttranscriptional silencing plays an important role in the early stage of germ cell development, then transcriptional silencing becomes important in later stages. In addition, intergenic and intronic retrotransposon sequences, in particular those containing the antisense L1 promoters, drove ectopic expression of nearby genes in both mutant spermatocytes, suggesting that retrotransposon silencing is important for the maintenance of not only genomic integrity but also transcriptomic integrity.

## Author summary

Retrotransposons are a class of transposable elements, of which mobility has mutagenic potential. Therefore, it is important to regulate the expression of retrotransposons for maintaining the genomic integrity. In male germ cells, DNA methylation and the piRNA system are thought to play roles in retrotransposon silencing. However, genome-wide DNA methylation is once erased (in primordial germ cells) and reestablished (in prospermatogonia) during development. In prospermatogonia, piRNAs guide *de novo* DNA methylation at some retrotransposons. To clarify the contribution of DNA methylation and the piRNA system to retrotransposon silencing in the course of male germ cell development, we analyzed DNA methylation and RNA expression in *Dnmt3l* and *Pld6* knockout mice, which are defective in *de novo* DNA methylation and piRNA biogenesis, respectively. Our results reveal that, in prospermatogonia, the piRNA system works mainly at the posttranscriptional level, and plays a more important role than does DNA methylation in retrotransposon silencing. However, DNA methylation becomes much more important in later stages when germ cells enter meiosis (in spermatocytes). We also found that hypomethylated retrotransposons can drive ectopic expression of nearby genes; therefore, their transcriptional silencing by DNA methylation is important for maintaining the transcriptomic integrity as well.

## Introduction

The mouse genome harbors millions of copies of transposable elements, the majority of which are retrotransposons. The retrotransposons include long terminal repeat (LTR) elements, long interspersed elements (LINEs), and short interspersed elements (SINEs) [1]. The LTR elements include endogenous retroviruses (ERVs). Some retrotransposons, such as the intracisternal A particle (IAP, which is an ERV) and LINE-1 (L1, a LINE), are active in transposition and have a potential to cause insertion mutations. Thus, retrotransposons present a threat to genomic integrity. In general, the expression of retrotransposons is epigenetically regulated at the transcriptional level by DNA methylation at CpG sites and by histone modifications, such as histone H3 methylation at lysine 9 (H3K9me). For example, a knockout (KO) mutation of *Dnmt1*, which encodes a maintenance-type DNA methyltransferase, causes derepression of IAPs in whole mouse embryos because of a passive loss of DNA methylation [2]. When DNA methylation is completely lost from embryonic stem cells (ESCs) by KO mutation of all three DNA methyltransferase genes (*Dnmt1*, *Dnmt3a*, and *Dnmt3b*), the expression of L1 elements, but not of IAP elements, is increased [3, 4]. Moreover, a KO mutation of *Setdb1/Eset*, which encodes an H3K9 methyltransferase, causes increased expression of LTR elements, such as IAP elements, in mouse ESCs and brain, but not in embryonic fibroblasts [4–6]. Thus, although both DNA methylation and histone modifications are important for silencing, the dominant mechanism differs among cell types and retrotransposons.

**Fig 1:**
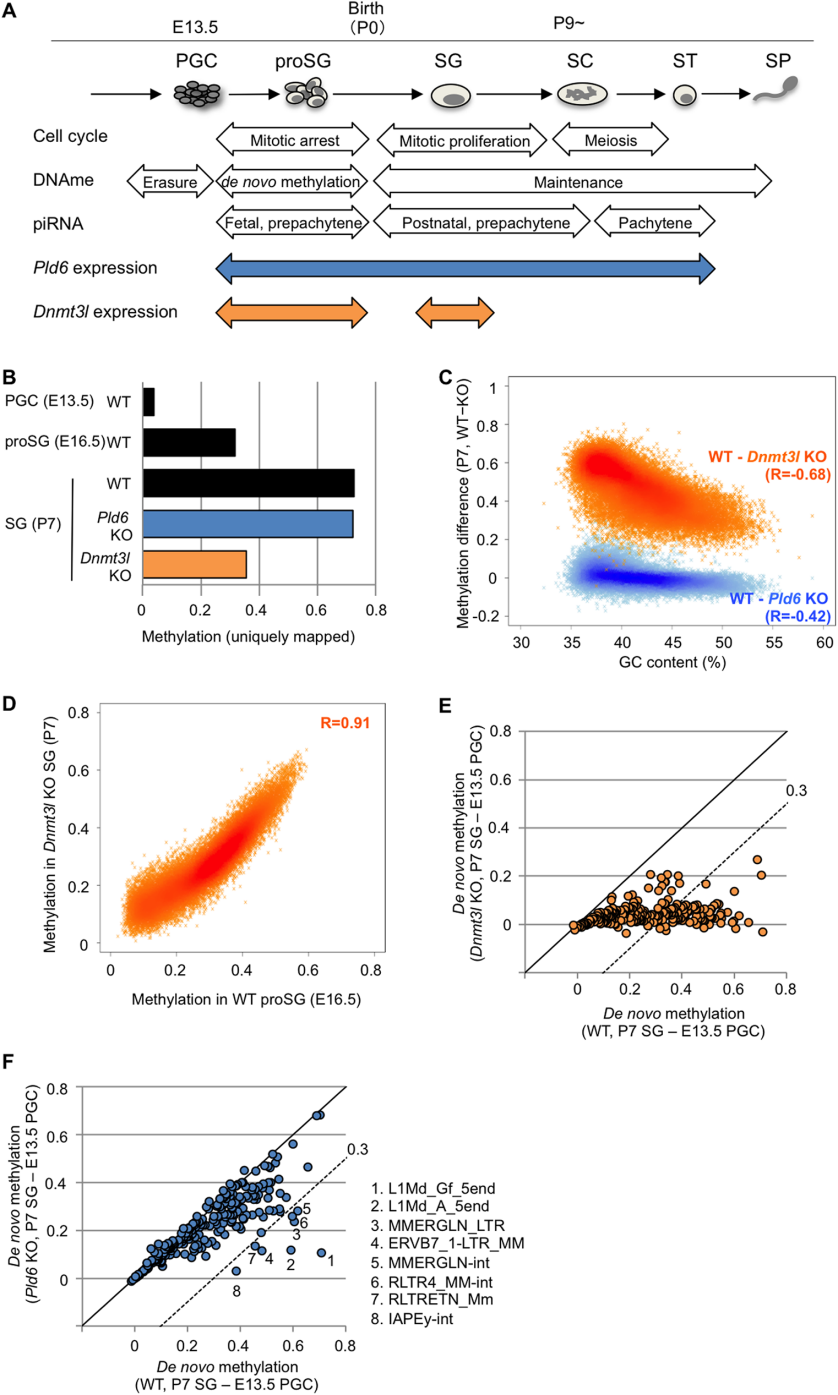
DNA methylation profiles in *Pld6* KO and *Dnmt3l* KO spermatogonia. (A) Schematic representation of male germ cell development with events related to the cell cycle, DNA methylation, and piRNA expression. The *Pld6* and *Dnmt3l* expression patterns are also shown. proSG, prospermatogonium; SG, spermatogonium; SC, spermatocyte; ST, spermatid; SP, spermatozoon. (B) Overall methylation levels at CpG sites. For WT E13.5 PGCs and E16.5 prospermatogonia, published data were used [32]. (C) Methylation differences between WT and KO spermatogonia and their relationship with GC content. The analysis was performed in 100 kb windows. Pearson’s *R* values are shown in parentheses. (D) Correlation between the methylation levels in *Dnmt3l* KO spermatogonia and those in WT E16.5 prospermatogonia. The analysis was performed in 100 kb windows. (E) Comparison of the extent of *de novo* methylation at individual retrotransposons in *Dnmt3l* KO and WT germ cells. The methylation levels detected in E13.5 PGCs were subtracted from those observed in P7 spermatogonia. Each spot represents one retrotransposon species. The dashed line denotes the following slope: y = x − 0.3. (F) Comparison of the extent of *de novo* methylation at individual retrotransposons in *Pld6* KO and WT germ cells. Details are as in (E).

During germ cell development, the DNA methylation profile changes dynamically [7] (Fig 1A). Initially, primordial germ cells (PGCs) have a low methylation level at embryonic day 13.5 (E13.5); subsequently, global *de novo* methylation occurs in mitotically arrested prospermatogonia, between E13.5 and the newborn stage (postnatal day 0 or P0). A few days after birth, the arrested cells resume mitosis, giving rise to spermatogonia, and, at around P9, they start to differentiate into spermatocytes and initiate meiosis. The global CpG methylation level established in late prospermatogonia is maintained throughout spermatogenesis, although there are local changes at regulatory elements [8, 9] (Fig 1A). *Dnmt3l* and *Dnmt3a*, which respectively encode a DNA methyltransferase-like protein and a *de novo* DNA methyltransferase, are highly expressed in prospermatogonia [10, 11]. KO mutations of *Dnmt3l* result in a failure in *de novo* methylation of the IAP and L1 promoters and the centromeric and pericentromeric repeats [12–14]. Moreover, *Dnmt3l* KO mice show increased expression of the IAP and L1 family members in postnatal testes [12, 15], suggesting the important role of this gene in retrotransposon silencing.

In prospermatogonia, PIWI-interacting RNAs (piRNAs) are generated by the actions of many proteins, including PLD6/MitoPLD/ZUC, PIWIL2/MILI, and PIWIL4/MIWI2 [16]. PLD6 is a phospholipase D/nuclease family protein that contributes to the generation of primary piRNAs by cleaving piRNA precursors [17–19]. PIWIL2 binds primary piRNAs and cleaves target RNAs that are complementary to the bound piRNAs. The cleaved RNAs are further processed to yield secondary piRNAs, which are used for another round of piRNA production via the so-called ping-pong cycle [20]. A large part of the piRNAs in mouse prospermatogonia is derived from retrotransposons and other transposable elements: therefore, the piRNA system is considered as a host defense system [21, 22]. In fact, the L1 and IAP families are derepressed in *Pld6* KO testes at P14 [19]. Interestingly, prospermatogonia from *Pld6* KO, *Piwil2* KO, and *Piwil4* KO mice fail to achieve *de novo* methylation at the L1 promoters [19, 20, 23–25], suggesting that piRNAs may guide *de novo* methylation [26]. Thus, the piRNA system likely silences retrotransposons at both the transcriptional and posttranscriptional levels.

In a previous study, we showed that DNA methylation is virtually unaffected at the IAP, MMERVK10C, and SINE B1 sequences in *Pld6* KO spermatogonia [27]. It was also reported that *Piwil2* KO mutants properly gain methylation at many retrotransposon sequences, with the exception of the L1 promoters [28, 29]. Thus, the piRNA-guided *de novo* methylation and, in turn, transcriptional silencing, may occur only at limited families of retrotransposons, such as the L1 family, in prospermatogonia. In *Mael* and *Hsp90aa1/Hsp90α* KO prospermatogonia, in which the amount of piRNA is severely reduced, the methylation of the L1 promoters is unchanged, whereas the L1 protein level is increased [30, 31], suggesting the presence of piRNA-mediated posttranscriptional regulation.

In the present study, we attempted to clarify the contributions of the piRNA system and DNA methylation to retrotransposon silencing during male germ cell development in greater detail. We performed whole-genome bisulfite shotgun sequencing and RNA sequencing (RNA-seq) in germ cells obtained from *Pld6* KO and *Dnmt3l* KO mice. Our results demonstrate an interesting shift from a posttranscriptional to a transcriptional mechanism as the major contributor to retrotransposon silencing during male germ cell development. We also show that retrotransposon silencing is important not only for genomic integrity, but also for transcriptomic integrity.

## Results

### *Pld6* contributes to *de novo* DNA methylation in a subset of retrotransposons

To examine the effect of *Pld6* KO and *Dnmt3l* KO mutations on *de novo* DNA methylation of retrotransposons, we performed whole-genome bisulfite shotgun sequencing in wild-type (WT), *Pld6* KO, and *Dnmt3l* KO spermatogonia that were collected by fluorescence-activated cell sorting (FACS) at postnatal day 7 (P7). Using uniquely mapped reads, we determined the average methylation level at CpG sites (the methylated cytosine calls divided by the sum of the methylated and unmethylated cytosine calls), which were 0.73, 0.72, and 0.36 (in fraction values) in WT, *Pld6* KO, and *Dnmt3l* KO spermatogonia, respectively (Fig 1B, S1 Fig). This suggests that, while *Dnmt3l* is important for *de novo* methylation at unique sequence regions, *Pld6* is dispensable for this process. Although the impact of the *Dnmt3l* mutation was global, GC-poor (or AT-rich) regions were more severely affected (Pearson’s *R* = −0.68) (Fig 1C, S2 Fig). These regions overlapped well with regions that were poorly methylated at E16.5 [32] in WT prospermatogonia (Fig 1D, S2 Fig), indicating that the *de novo* methylation of the GC-poor regions is more dependent on *Dnmt3l* and occurs later in normal development.

Next, the DNA methylation levels of relatively young (i.e., mouse-specific) retrotransposons (n = 263) were determined in each genotype by mapping the reads onto their consensus sequences in the RepeatMasker library (for some retrotransposons, the sequences were divided into portions; e.g., 5end, orf2, and 3end). To determine the extent of *de novo* methylation in individual retrotransposons, we subtracted the methylation levels determined in E13.5 WT PGCs (onset of *de novo* methylation) [32] from those determined in P7 spermatogonia (after *de novo* methylation). In *Dnmt3l* KO spermatogonia, almost all retrotransposons were severely affected (Fig 1E, S3 and S4 Figs and S1 Table), whereas the *Pld6* mutation had a lesser impact (Fig 1F, S3 Fig and S1 Table). The methylation levels of most retrotransposons in *Pld6* KO spermatogonia were very similar to those observed in *Piwil2* KO spermatogonia (S5 Fig) [28, 29], and only a subset of retrotransposons, including L1Md_A_5end and L1Md_Gf_5end (the 5′ regions of A- and G_F_-type L1, respectively), were severely affected (≥0.3).

### *Pld6* contributes to the silencing of the L1 and IAP families in prospermatogonia, independent of DNA methylation

**Fig 2:**
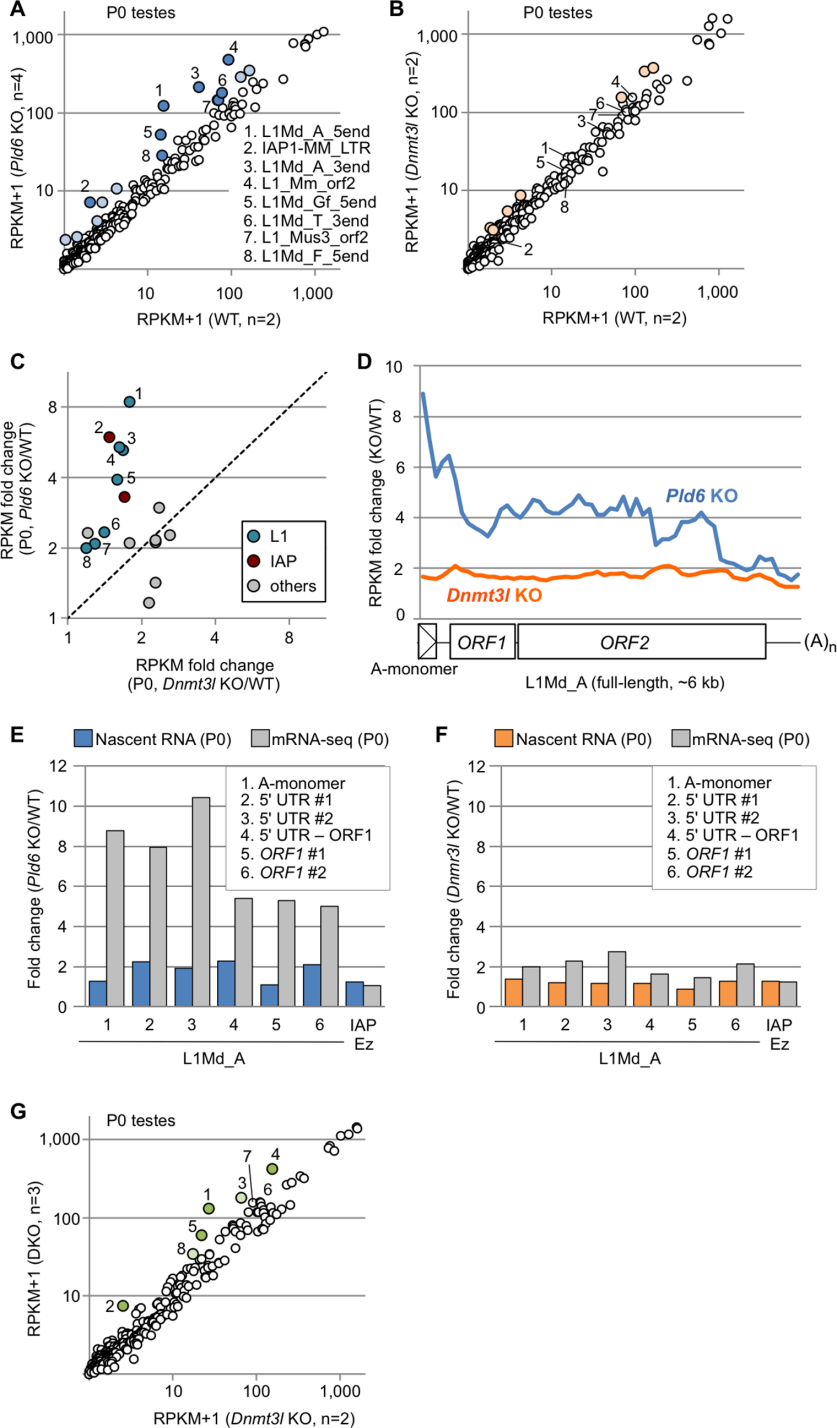
Retrotransposon expression in *Pld6* KO and *Dnmt3l* KO newborn testes. (A) Expression of individual retrotransposons in *Pld6* KO and WT testes. Those showing increased expression (>2-fold) in KO testes are colored (*P* < 0.05, blue spots with numbers 1–8; *P* > 0.05, light blue). The numbered retrotransposons are marked by the same numbers in B, C, and G. (B) Retrotransposon expression in *Dnmt3l* KO and WT testes. The light-orange spots represent retrotransposons showing increased expression in KO testes (>2-fold, *P*>0.05). (C) Expression of individual retrotransposons in *Pld6* KO and *Dnmt3l* KO testes relative to WT testes. Only those showing a >2-fold increase in either mutants are shown. Members of the L1 and IAP families are indicated in blue and red, respectively. (D) Fold change in L1Md_A expression across the consensus sequence in *Pld6* KO and *Dnmt3l* KO testes relative to WT testes. Reads were counted in nonoverlapping 100-bp windows. The reference sequence had one A-monomer (position 1–207). (E) Fold changes in nascent L1Md_A RNA expression in *Pld6* KO prospermatogonia compared with WT prospermatogonia (blue bars). The experiments were performed using eight biological replicates. Fold changes in steady-state RNA level are shown for comparison (gray bars). PCR regions 1–6 are indicated in the inset. (F) Fold changes in nascent L1Md_A RNA expression in *Dnmt3l* KO prospermatogonia compared with WT prospermatogonia (orange bars). The experiments were performed using five and two biological replicates for WT and KO cells, respectively. Fold changes in steady-state RNA level are also shown (gray). (G) Expression of individual retrotransposons in double KO and *Dnmt3l* KO testes. Retrotransposons showing increased expression (>2-fold) in double KO testes are colored (*P* < 0.05, green; *P* > 0.05, light green). DKO, double KO.

To determine whether the *Pld6* and *Dnmt3l* mutations affect the RNA levels of retrotransposons in prospermatogonia, we performed deep sequencing of polyA(+) RNAs (mRNA-seq) with strand discrimination in newborn (P0) testes, where prospermatogonia are the only germ cell component. (Note that LINE and LTR elements have a polyadenylation signal [33, 34] and their RNAs are polyA(+).) The mapping of the reads to the consensus sequences revealed that the expression of the L1 and IAP family members was increased by >2-fold in *Pld6* KO testes (*P* < 0.05, *t* test; Fig 2A, S1 Table). In *Dnmt3l* KO testes, the expression of only a small subset of retrotransposons was increased by >2-fold, albeit with little significance (*P* > 0.05 for all; Fig 2B, S1 Table). Thus, the *Pld6* mutation had a greater impact on retrotransposon silencing than did the *Dnmt3l* mutation (Fig 2C), despite the observation that the *Dnmt3l* mutation affected *de novo* methylation more severely (Fig 1E and 1F). These results suggest that *Pld6* silences retrotransposons predominantly via a mechanism that is independent of methylation.

The mapping of RNA reads to the full-length L1Md_A sequence revealed that the increase in expression was most prominent in the 5′ region (up to 10-fold) in *Pld6* KO testes (Fig 2D, blue line), whereas it was constant (1.8-fold) throughout the L1Md_A sequence in *Dnmt3l* KO testes (Fig 2D, orange line). As the RNA-seq libraries were constructed from polyA(+) RNAs, our results suggest that full-length plus near-full-length L1Md_A RNAs are more abundant, thus RNA cleavage is less frequent in *Pld6* KO testes. When both cleaved and uncleaved RNAs were together measured in total RNA from P0 testes by random priming followed by quantitative PCR (qRT-PCR), a uniform 1.7- to 2.8-fold increase was observed along the L1Md_A sequence in *Pld6* KO testes (S6 Fig). Next, we quantified the nascent RNAs of L1Md_A in newborn testes by ethynyl uridine labeling, purification, and subsequent qRT-PCR and found that transcription was increased only 1.1- to 2.3-fold in *Pld6* KO testes (Fig 2E). Thus, the higher expression detected in these regions by RNA-seq (5- to 10-fold, Fig 2E) was more likely caused by increased RNA stability (less RNA cleavage) rather than increased transcription. In contrast, in *Dnmt3l* KO testes, the increase in the nascent RNAs was not much different from that observed in the steady-state RNA by RNA-seq (Fig 2F), suggesting regulation at the transcriptional level. Moreover, the introduction of the *Pld6* mutation in addition to the *Dnmt3l* mutation further increased the RNA levels of the L1 and IAP family members (compare *Pld6*/*Dnmt3l* double KO with *Dnmt3l* KO; Fig 2G, S7 Fig and S1 Table). This suggests that, in prospermatogonia, retrotransposon silencing by *Pld*6 does not require *Dnmt3l*, is largely independent of DNA methylation, and, thus, perhaps occurs through piRNA-guided RNA cleavage.

**Fig 3:**
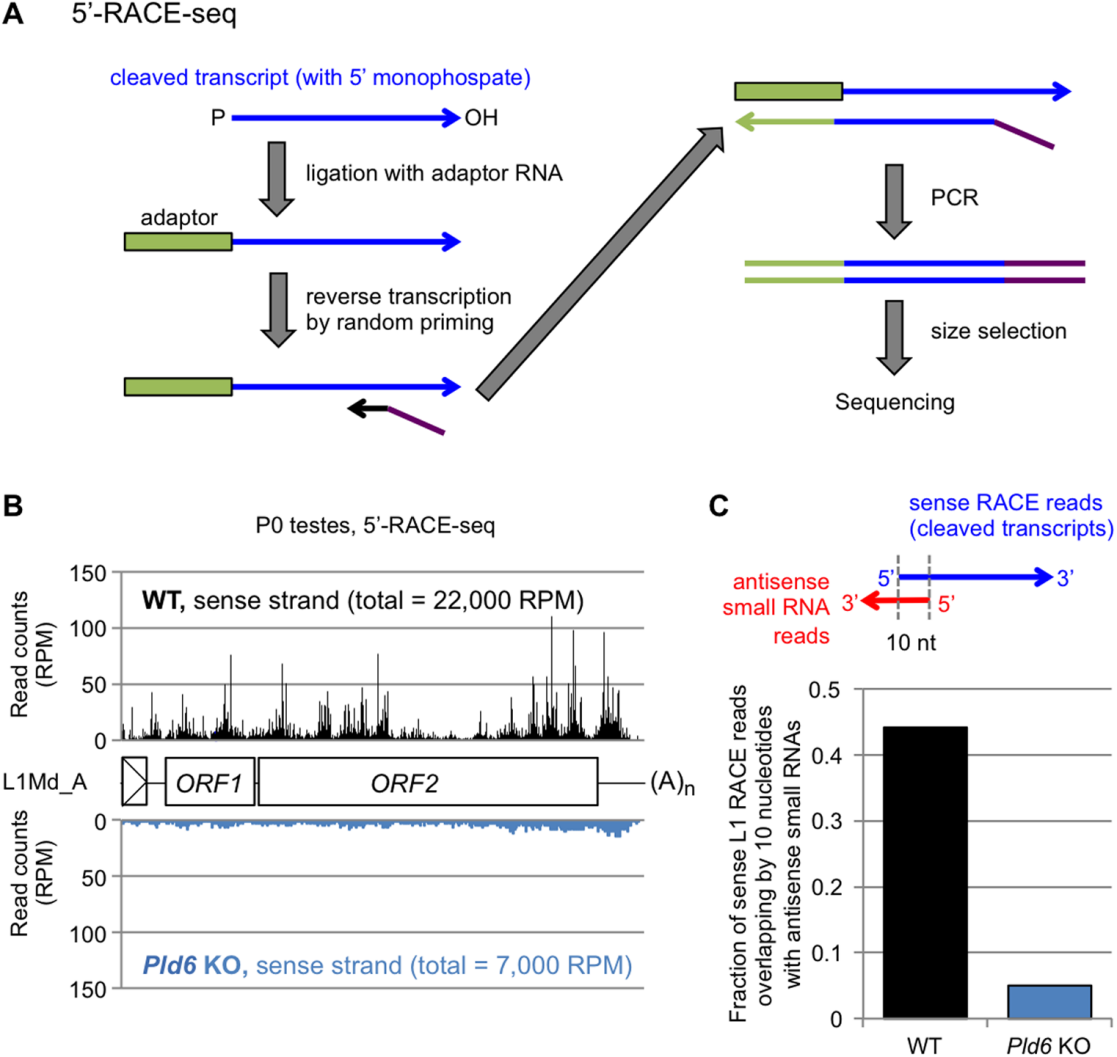
*Pld6*-dependent degradation of L1 RNAs. (A) Library preparation for 5'-RACE-seq. RNAs with 5' monophosphate (blue arrow) are ligated to an RNA adaptor (green box), allowing selective amplification of cleaved RNAs for subsequent reverse transcription and PCR. (B) 5'-RACE-seq reads of WT (top, black) and *Pld6* KO testes (bottom, blue) at P0 were mapped to the L1Md_A consensus sequence. Total read counts (in RPM, reads per million mapped reads) are shown in parenthesis. (C) Fraction of 5'-RACE-seq reads (L1 sense strand) in P0 testes showing a 10-nt complementarity with an antisense piRNA.

To evaluate the role of piRNA-guided RNA cleavage in retrotransposon silencing in prospermatogonia, we profiled the piRNAs (24- to 33-nt small RNAs) from WT and *Pld6* KO testes at P0 by small RNA sequencing. The profile of retrotransposon-derived piRNAs in WT P0 testes was very similar to that in E16.5 testes [31] (R = 0.95), with 45% of them being derived from the L1 family (S1 Table). In *Pld6* KO P0 testes, the retrotransposon-derived piRNAs were severely reduced (S8 Fig), as was observed in *Pld6* KO E16.5 testes [19]. Importantly, retrotransposons with a large drop (>1,000 RPM) in the amount of antisense piRNAs in *Pld6* KO testes showed a significant increase in RNAs in the mutants (S8 Fig and S1 Table). Next, we tried to identify and quantify the cleaved L1 RNAs in WT and *Pld6* KO testes by 5'-RACE sequencing (5'-RACE-seq) (Fig 3). The method includes RNA adaptor ligation where only cleaved RNAs with a 5' monophosphate group can accept the adapter: non-cleaved RNAs with a triphosphorylated or a capped 5' end are not reactive [35, 36] (Fig 3A). We mapped the 5'-RACE-seq reads to the full-length L1Md_A sequence and to the whole genome and revealed a number of RNA cleavage sites throughout the L1Md_A sequence in WT testes at P0 (Fig 3B). Importantly, about half of the cleaved RNAs showed a 10-nt complementarity with an antisense piRNA at their 5’ portions (Fig 3C), a feature of the piRNA-guided RNA cleavage. The fraction of L1-derived RNA fragments in the total cleaved RNAs (mapped to the whole genome) was reduced 3.1-fold in the *Pld6* KO testes (Fig 3B). Given that L1 transcription increased 2-fold in the mutant testes (Fig 2E), the above results suggest that the observed L1 RNA cleavage largely depended on *Pld6*. Thus, our findings suggest that the piRNA-guided RNA cleavage, which directly or indirectly involves *Pld6*, is important for retrotransposon silencing in prospermatogonia.

### DNA methylation has an important role in retrotransposon silencing during meiosis

Next, we studied the impact of the *Pld6* KO and *Dnmt3l* KO mutations on retrotransposon silencing in meiotic germ cells. WT testes at P21 contained all of the premeiotic (spermatogonia), meiotic (spermatocytes), and postmeiotic (spermatids) germ cells (Fig 1A), as confirmed by FACS profiling (S9 Fig). However, only spermatogonia and spermatocytes (at the preleptotene, leptotene, and zygotene stages) were present in *Pld6* KO and *Dnmt3l* KO testes (S9 Fig), because of the cell death that occurs at the late zygotene stage [14, 19]. Thus, we collected leptotene/zygotene (L/Z) spermatocytes by FACS and performed polyA(+) RNA-seq.

**Fig 4:**
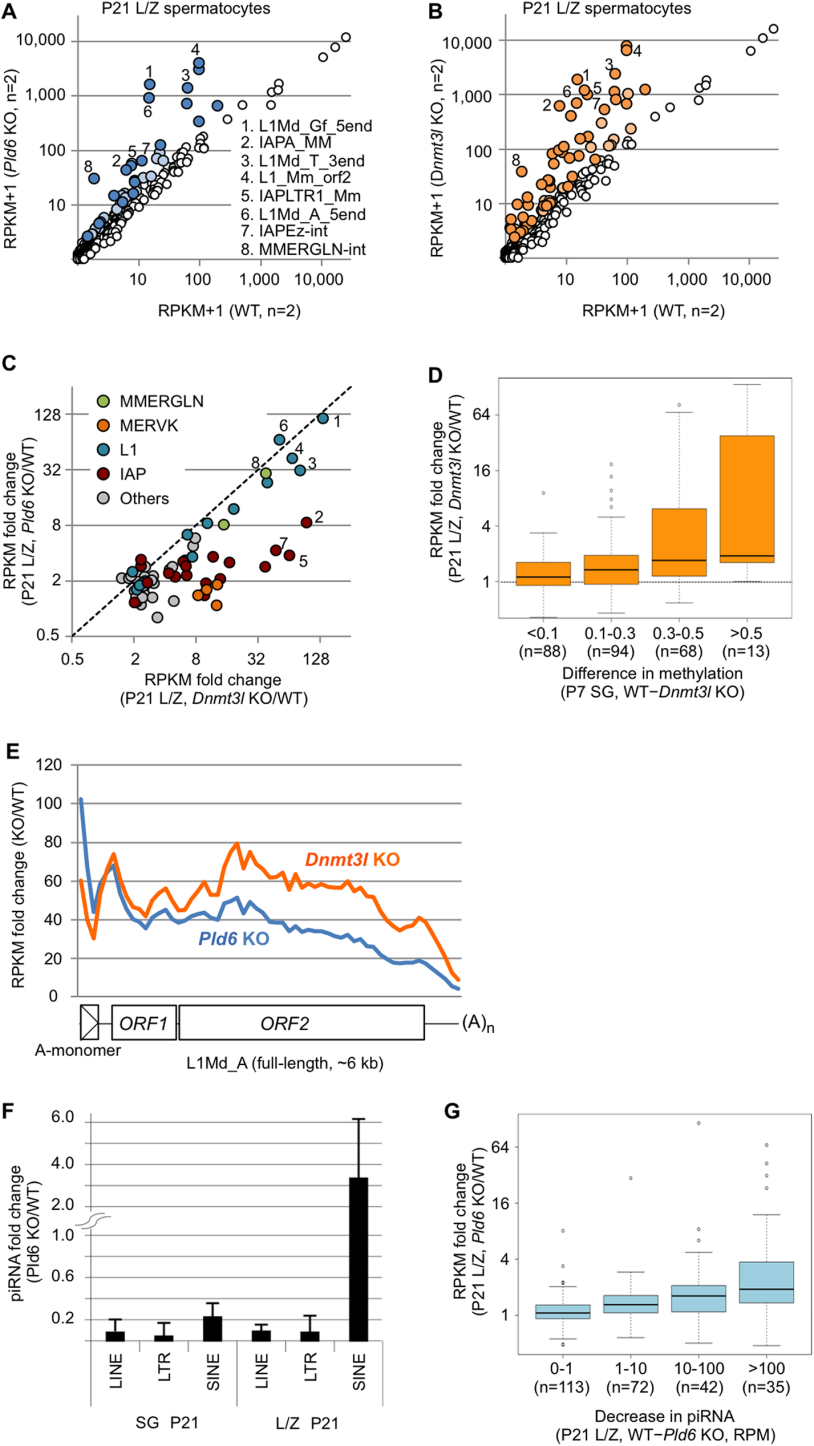
Retrotransposon expression in *Pld6* KO and *Dnmt3l* KO spermatocytes. (A) Expression of individual retrotransposons in *Pld6* KO and WT spermatocytes. Those showing increased expression (>2-fold) in KO spermatocytes are colored (*P* < 0.05, blue; *P* > 0.05, light blue). The retrotransposons showing the largest fold increases are numbered 1–8 and labeled using the same numbers in B and C. (B) Retrotransposon expression in *Dnmt3l* KO and WT spermatocytes. Those showing increased expression (>2-fold) in KO spermatocytes are colored (*P* < 0.05, orange; *P* > 0.05, light orange). (C) Expression of individual retrotransposons in *Pld6* KO and *Dnmt3l* KO spermatocytes relative to WT spermatocytes. Only those showing a >2-fold increase in either mutant are shown. Members of the MMERGLN, MERVK10/RLTR10, L1, and IAP families are shown in green, orange, blue, and red, respectively. (D) Relationship between the increase in expression in *Dnmt3l* KO spermatocytes and the decrease in methylation in *Dnmt3l* KO spermatogonia. Retrotransposons are grouped according to the degree of decreased methylation in *Dnmt3l* KO spermatogonia relative to WT spermatogonia. Box plots show the medians (solid line), the first and third quartiles (bottom and top of the boxes), values of 1.5-fold the interquartile range from the first and third quartiles (whiskers), and outliers (circles). (E) Fold change in L1Md_A RNA expression across the consensus sequence in *Pld6* KO and *Dnmt3l* KO spermatocytes relative to WT spermatocytes. Details are as in Fig 2D. (F) Fold changes in the abundance of piRNAs derived from retrotransposons in *Pld6* KO spermatogonia and *Pld6* KO L/Z spermatocytes relative to their WT counterparts. Error bars indicate the standard deviation. (G) The relationship between the increase in expression of retrotransposons and the decrease in the abundance of the corresponding antisense piRNAs in *Pld6* KO spermatocytes relative to WT spermatocytes. Retrotransposons are grouped according to the degree of decrease in antisense piRNA abundance in *Pld6* KO spermatocytes. The box plot features are as described in Fig 3D. RPM, reads per million reads.

The results of this experiment showed that the *Pld6* KO and *Dnmt3l* KO mutations caused a >2-fold increase in the expression of 21 and 46 retrotransposons, respectively (*P* < 0.05; Fig 4A and 4B, S1 Table). In particular, the expression of some L1 family members was increased by >30-fold in both mutants and the expression of some IAP, MERVK, and MMERGLN family members was increased by >10-fold in the *Dnmt3l* mutants (Fig 4C). Thus, both mutations had a greater impact on retrotransposon silencing in L/Z spermatocytes than they did in prospermatogonia (P0 testes) (compare Figs 4A and 4B and 2A and 2B). However, the *Dnmt3l* mutation had a greater impact than did the *Pld6* mutation in spermatocytes (Fig 4A and 4B). Thus, the relative importance of *Pld6* and *Dnmt3l* in retrotransposon silencing was reversed between the stages (compare Figs 2C and 4C). Moreover, in contrast to the observations from P0 testes (S8 Fig), the increase in expression observed in *Dnmt3l* KO spermatocytes correlated well with the decrease in methylation detected in *Dnmt3l* KO spermatogonia (Fig 4D). In addition, the expression levels of the L1Md_A, L1Md_Gf, and MMERGLN family members were similar between *Pld6* KO and *Dnmt3l* KO spermatocytes (Fig 4C), which agrees well with the similar hypomethylation observed in their promoters (Fig 1E and 1F, S3 Fig and S1 Table). The expression levels of the IAP and MERVK family members were higher in *Dnmt3l* KO spermatocytes (Fig 4C) and correlated well with the differences in methylation observed between the mutants (S3 Fig and S1 Table). Comparisons of the RNA-seq data of *Pld6* KO spermatocytes with those of *Piwil2* and *Piwil4* KO testes at P10 [29] revealed that similar sets of retrotransposons were derepressed in the *Pld6* and *Piwil2* KO mutants (S5 Fig). In contrast, only retrotransposons with reduced DNA methylation showed increased expression in *Piwil4* KO testes (S5 Fig). For example, the *Piwil4* KO mutation reduced the methylation level of L1Md_Gf_5end, but not L1Md_A_5end, and consistently, the expression of L1Md_Gf_5end, but not L1Md_A_5end, was elevated in the *Piwil4* KO testes (S5 Fig). All of these results further support the important role of DNA methylation in retrotransposon silencing in L/Z spermatocytes.

### A posttranscriptional mechanism also plays a role in retrotransposon silencing during meiosis

Despite the importance of DNA methylation during meiosis, it was reported that PIWIL2 regulates L1 at the posttranscriptional level in spermatocytes [37]. In addition, the expression of some retrotransposons, such as IAPA_MM, was increased in *Pld6* KO mutants without a large decrease in DNA methylation (S1 Table). Therefore, we conducted a couple of analyses to examine the contribution of piRNA-guided RNA cleavage to retrotransposon regulation in spermatocytes. First, we mapped the RNA-seq reads obtained from *Pld6* KO spermatocytes to the full-length L1Md_A sequence and found that the increase in expression was highest in the 5′ region (up to 100-fold) (Fig 4E, blue line) and declined toward the 3′ end, resembling the pattern observed in P0 testes (Fig 2D). This suggests that RNA cleavage contributes to silencing, at least partly, in spermatocytes.

Second, we profiled small RNAs in FACS-purified L/Z spermatocytes from P21 WT and *Pld6* KO testes by deep sequencing, and detected a severe loss of piRNAs derived from LINE and LTR elements, but not of those derived from SINE elements, in *Pld6* KO spermatocytes (Fig 4F), which was consistent with our previous data from P10 testes [19]. Importantly, the degree of decrease in piRNA abundance in *Pld6* KO spermatocytes correlated well with the degree of increase in target RNA abundance (Fig 4G), which is consistent with the direct involvement of piRNAs in posttranscriptional silencing.

### Retrotransposon activation disrupts the integrity of the transcriptome

**Fig 5:**
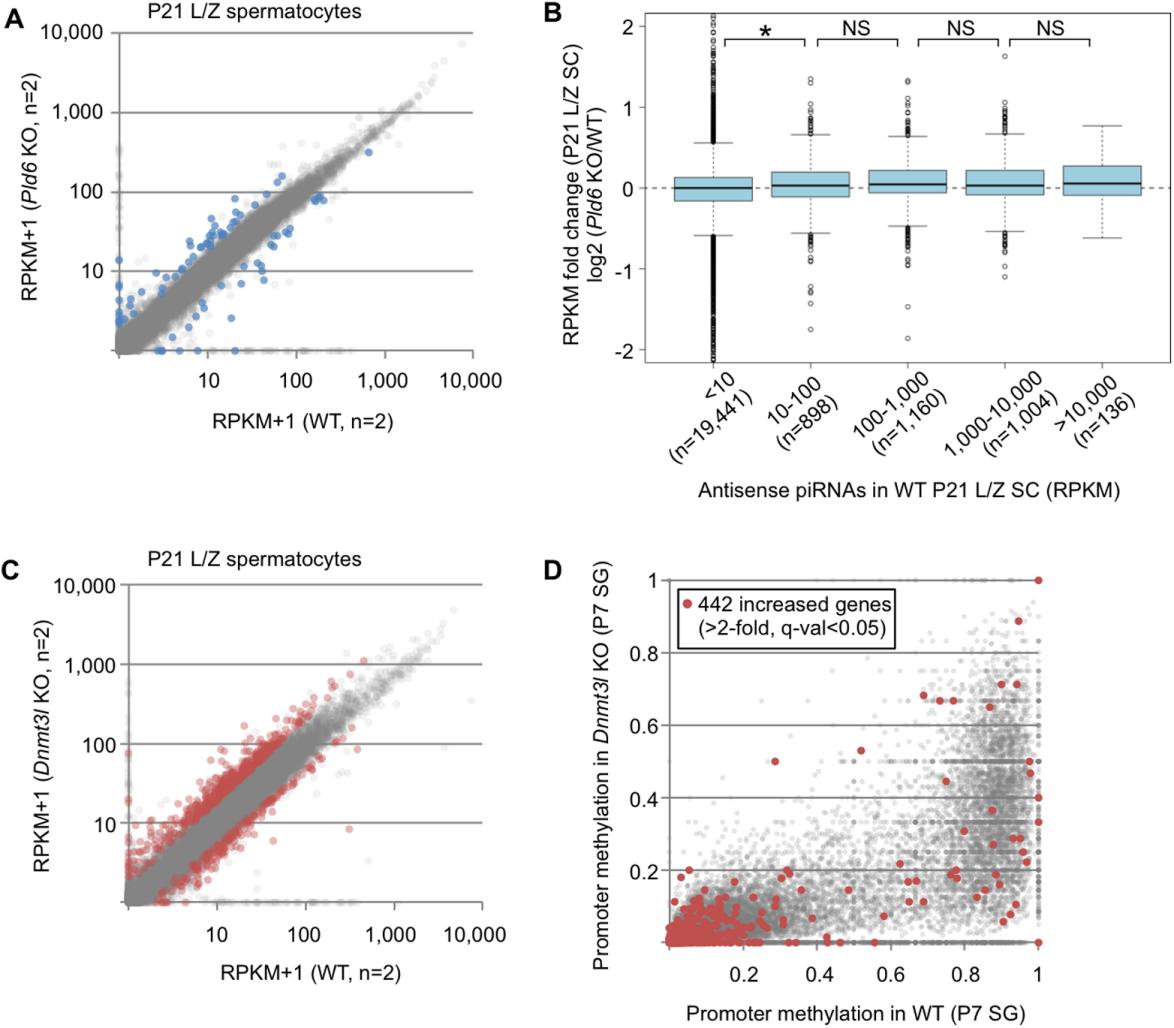
RefSeq gene expression profiles in *Pld6* KO and *Dnmt3l* KO spermatocytes. (A) Expression of protein-coding and noncoding genes (n = 22,771, excluding miRNAs, small nucleolar RNAs, and rRNAs) in *Pld6* KO and WT spermatocytes. Genes showing increased or decreased expression in *Pld6* KO spermatocytes are colored in blue (>2-fold or <1/2, q-value < 0.05). (B) Relationship between the increase in RefSeq gene expression in *Pld6* KO spermatocytes and the abundance of piRNAs antisense to the genes in WT spermatocytes. Genes are grouped according to the abundance of antisense piRNAs (RPM). The box plot features are as described in Fig 3D. An asterisk indicates that the difference is significant (*P* < 0.05, *U* test). NS, not significant. (C) Expression of protein-coding and noncoding genes in *Dnmt3l* KO and WT spermatocytes. Genes showing increased or decreased expression in *Dnmt3l* KO spermatocytes are colored in red (>2-fold or <1/2, q-value < 0.05). (D) Promoter methylation levels in *Dnmt3l* KO and WT spermatogonia. The red spots indicate the promoters of genes showing increased expression in *Dnmt3l* KO spermatocytes (>2-fold, q-value < 0.05).

We then examined whether the RefSeq expression profile (transcriptome) is affected by the *Pld6* KO and *Dnmt3l* KO mutations. In *Pld6* KO L/Z spermatocytes, 41 and 32 genes showed increased (>2-fold) and decreased (<1/2) expression, respectively (q-value < 0.05, calculated by Cuffdiff; Fig 5A, S2 Table). In contrast to round spermatids, in which many RefSeq genes are directly regulated by piRNAs antisense to the genes [38], only a weak correlation was observed between the changes in gene expression in *Pld6* KO spermatocytes and the abundance of antisense piRNAs in WT spermatocytes (Fig 5B). Thus, the altered expression did not appear to involve piRNAs against the RefSeq genes themselves. In *Dnmt3l* KO spermatocytes, 442 and 184 genes, respectively, showed increased (>2-fold) and decreased (<1/2) expression (Fig 5C, S2 Table). Thus, 10 times more RefSeq genes were upregulated in *Dnmt3l* KO spermatocytes compared with *Pld6* KO spermatocytes. Only a small proportion of the upregulated genes exhibited significantly decreased levels of promoter methylation in *Dnmt3l* KO spermatogonia (Fig 5D), suggesting that promoter methylation of the genes themselves is largely irrelevant. In P0 testes, a smaller number of genes were affected compared with that observed in L/Z spermatocytes: 50 and 143 genes were affected (>2-fold and <1/2, q-val < 0.05) in *Pld6* KO and *Dnmt3l* KO mutants, respectively, and antisense piRNAs and promoter methylation seemed irrelevant (S10 Fig).

**Fig 6:**
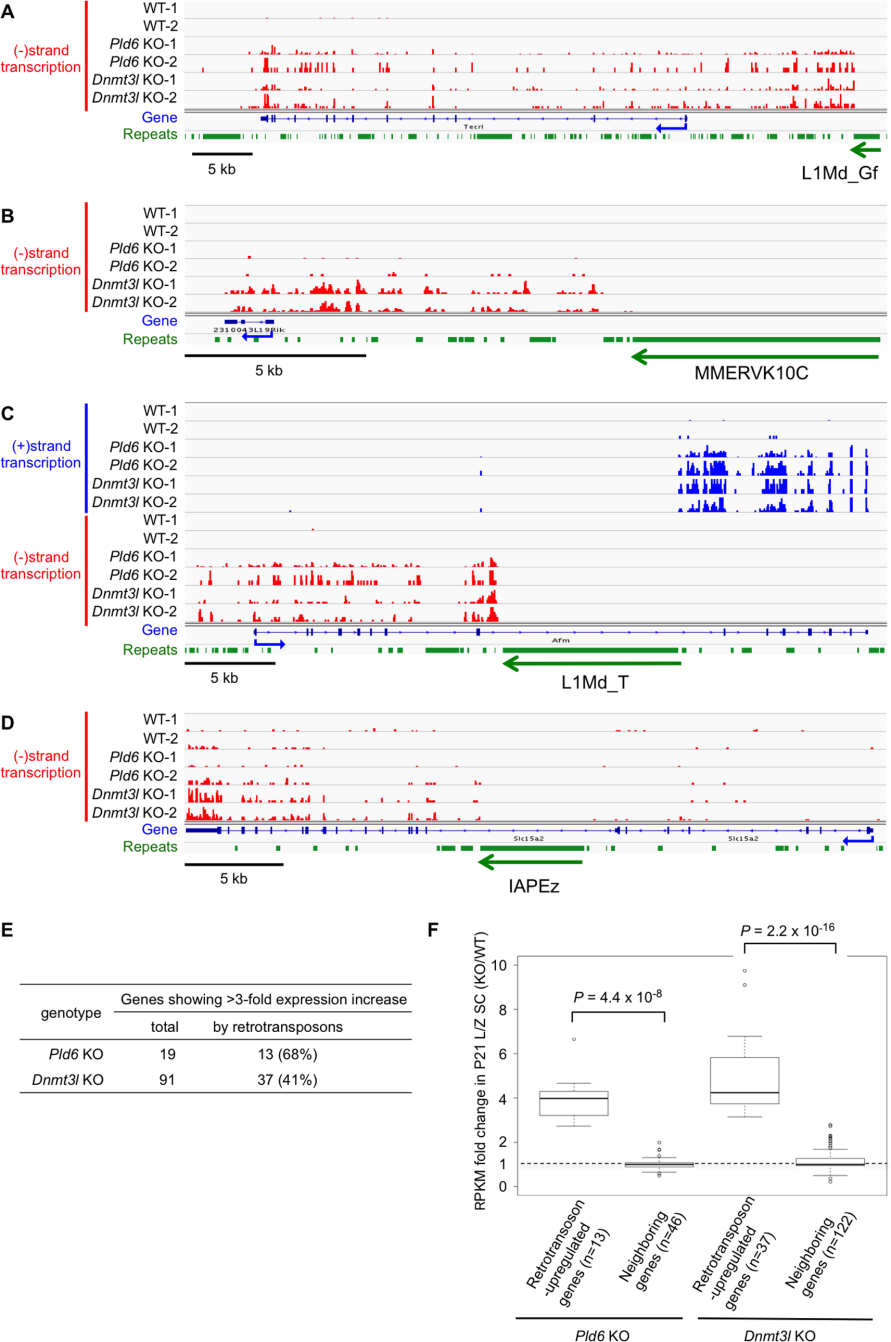
Retrotransposons induce neighboring genes in *Pld6* KO and *Dnmt3l* KO spermatocytes. (A–D) Integrative Genomics Viewer screenshots are shown for *Tecrl* driven by an upstream L1Md_Gf copy (A), *2310043L19Rik* driven by an upstream MMERVK10C copy (B), *Caps2* driven by an intronic (antisense) L1Md_T copy (C), and *Slc15a2* driven by an intronic IAP (D). The y-axis shows the read density: the upper limit corresponds to 1.0 RPM (reads at each nucleotide position per million mapped reads) in all panels. Reads representing plus-strand (blue) and minus-strand (red) transcripts are shown separately. Reads matching repeat regions are removed because of the uncertainty in their origin. KO-1 and -2 and WT-1 and -2 indicate biological replicates. The orientation of the retrotransposon affecting the gene is indicated at the bottom of each panel. (E) Number of genes with a >3-fold increase in expression in *Pld6* KO and *Dnmt3l* KO. See supplementary S3 and S4 Tables for the list of these genes. (F) Expression of retrotransposon-driven genes and neighboring genes (within 100 kb) in *Pld6* and *Dnmt3l* KO spermatocytes. *P* values determined using the *U* test are shown.

Retrotransposons may provide alternative promoters for neighboring genes, and such fusion transcripts have been observed in *Setdb1* KO ESCs and PGCs [5, 39]. Although we did not find direct evidence for fusion transcripts in our single-end RNA-seq data, *Pld6* KO and D*nmt3l* KO spermatocytes contained derepressed L1, IAP, and MERVK copies whose transcription appeared to extend beyond their 3’ end and reach the nearby RefSeq genes. For example, an ectopic activation of *Tecrl* in both mutants was accompanied by derepression of an L1Md_Gf copy located 35-kb upstream of this gene and the region between them (Fig 6A). Similarly, a MMERVK10C copy appeared to drive its downstream gene in *Dnmt3l* KO spermatocytes, with a lesser effect observed in *Pld6* KO spermatocytes (Fig 6B). We also found that the antisense promoter [40] of a full-length copy of L1Md_T (T_F_-type L1), located in the eighth intron of *Afm* in an antisense orientation, likely drove the transcription of downstream exons in both mutants (Fig 6C). In another example, an intronic IAP copy in *Slc15a2* appeared to drive the transcription of downstream exons in both mutants, but at a much lower level in *Pld6* KO spermatocytes (Fig 6D).

Thus, of the 19 genes that exhibited a >3-fold increase in expression in *Pld6* KO spermatocytes, 13 (68%) were accompanied by an activated L1 (11 genes) or RLTR10 (two genes) copy (*P* = 4.4 × 10^−7^, Fisher’s exact test; Fig 6E, S3 Table). Of the 91 genes that exhibited a >3-fold increase in expression in *Dnmt3l* KO spermatocytes, 37 (41%) were accompanied by an activated L1 (23 genes), IAP (seven genes), MERVK10/RLTR10 (six genes), or MMERGLN (one gene) copy (*P* = 2.2 × 10^−16^; Fig 6E, S4 Table). Of the remaining 54 genes, nine showed a large decrease (<0.3) in promoter methylation in *Dnmt3l* KO spermatocytes. These results demonstrate that, in spermatocytes, the derepression of retrotransposons disrupts the integrity of the transcriptome by generating fusion transcripts. In contrast, we did not find such fusion transcripts in P0 testes of either mutants, presumably because of the lower levels of derepression of retrotransposons at this stage.

It is possible that the higher expression of the RefSeq genes observed here resulted from regional activation, rather than from the derepression of individual retrotransposons. However, regional activation is unlikely because we did not observe increased expression of the neighboring RefSeq genes (<100 kb) (Fig 6F). We further examined whether the presence or absence of retrotransposons affects the induction of the RefSeq genes using strain hybrids. As some of the retrotransposon copies that drive the nearby genes were absent from the genome of the MSM/Ms strain [41] (S11 Fig), we generated *Pld6* KO and *Dnmt3l* KO mice in the F1 hybrid background (MSM/Ms × C57BL/6J). PolyA(+) RNA-seq analyses of L/Z spermatocytes using single-nucleotide polymorphisms revealed that the RefSeq alleles that are activated in both mutants are almost exclusively of C57BL/6J origin (S11 Fig). These results strongly suggest that the increased gene expression results from the derepression of nearby retrotransposons.

## Discussion

Robust silencing of retrotransposons is most likely achieved by a combination of DNA methylation, histone modifications, and small-RNA-mediated RNA degradation in mammalian cells. Among these mechanisms, DNA methylation is predominant in most cell types [2, 3, 12, 42]. Previous studies suggest that piRNAs guide the de novo methylation of some retrotransposons in prospermatogonia and that the increased expression of retrotransposons in piRNA-deficient germ cells may be caused by the failure in *de novo* methylation [20, 23–25]. However, in these studies, the expression was studied only in postnatal testes at P10 or later, and not in prospermatogonia. The present study showed that the loss of methylation caused by a *Dnmt3l* mutation did not severely affect the expression of any retrotransposons in newborn prospermatogonia, and that the *Pld6* mutation had a greater impact than did the *Dnmt3l* mutation (Fig 2); therefore, it is likely that posttranscriptional silencing is the primary strategy used for retrotransposon silencing in this cell type. In E13.5 PGCs, many retrotransposons are in fact active, but their expression levels decrease in E16.5 prospermatogonia [28], in which *de novo* methylation has not yet been completed (Fig 1B, S4 Fig). Of note, *de novo* methylation is especially delayed in retrotransposon-rich, GC-poor genomic regions at this stage (S2 Fig). Instead, the decline in retrotransposon expression appears to coincide with the accumulation of PIWI proteins and piRNAs [20, 28, 43, 44]. It should be noted that prospermatogonia are mitotically arrested: after the activation of retrotransposons, their RNA can persist without dilution via cell division. Thus, RNA cleavage guided by piRNAs would be beneficial to the host. Mammalian oocytes, which remain in the meiotic prophase for a long period and maintain a low methylation level [7], also use this strategy, and defects in the piRNA system in oocytes cause increased expression of some retrotransposons [45].

In contrast, DNA methylation is more important than RNA cleavage for retrotransposon silencing in spermatocytes. Decreased methylation had a great impact on the silencing of many retrotransposons, even in the presence of an intact piRNA system (Fig 4). It should be noted that the increased expression of specific retrotransposons (i.e., L1 and MMERGLN) observed in *Pld6* KO spermatocytes correlates well with the failure in *de novo* methylation. Similarly, disruption of *Piwil4* (expressed only in the fetal stage) results in a severe decrease in L1Md_Gf methylation and in an increase in its expression in postnatal testes [23]. In contrast, the same mutation does not affect methylation or expression of L1Md_A and IAP [23, 29]. These results indicate that the methylation pattern established in prospermatogonia has a long-term effect on retrotransposon silencing. The observed switch in the relative contribution of the different silencing mechanisms during germ cell development seems reasonable because methylation can be more easily maintained in postreplicative meiotic cells, despite the presence of dynamic histone modification changes and replacements. However, we note that piRNA-guided RNA cleavage did play a role in spermatocytes, which was consistent with previous work [37].

It should be noted that the ETn, MusD, and MERVL families, the transcriptional competence of which has been demonstrated in other cells [46–48], are not activated strongly in either *Pld6* KO or *Dnmt3l* KO mutants. Therefore, other mechanisms should compensate for the lack of piRNAs or DNA methylation. H3K9me3 is a likely candidate, because KO mutations of *Setdb1*, an enzyme that is responsible for this modification, increase the expression of some LTR elements, including the IAP, MMERVK10C, and ETn families, in ESCs and PGCs [4, 49]. In contrast, a role for H3K9me2 in this process is unlikely as its level is very low in PGCs and undifferentiated spermatogonia [50, 51]. Another candidate is H4R3me2, because a KO mutation of its responsible enzyme, PRMT5, leads to the activation of retrotransposons in PGCs [52]. However, PRMT5 has a large number of targets for arginine methylation, including PIWIL4, PIWIL2, and TRIM28/KAP1 [53]; thus, the manner via which this enzyme contributes to retrotransposon silencing needs to be clarified. In addition to the repressive chromatin modifications and the piRNA system, specific transcriptional activators and/or repressors may be involved here. For example, the L1 family can be repressed by ZBTB16/PLZF and SOX2 and activated by LEF1 [54–56]. ZBTB16, a factor involved in L1 silencing in testes [55], is expressed in prospermatogonia and undifferentiated spermatogonia, but not in spermatocytes [57], suggesting that a combinatorial loss of this repressor and methylation may allow L1 expression in spermatocytes. Expression of SOX2 is high in PGCs, but very low in E15.5 prospermatogonia [58], and its role in retrotransposon silencing in germ cells remains to be explored.

Finally, the derepression or activation of retrotransposons affected RefSeq genes in *Pld6* KO and/or *Dnmt3l* KO spermatocytes, thus disrupting the integrity of the transcriptome (Fig 6). The L1 family is the most predominant retrotransposon species that affected nearby genes. In particular, the antisense promoter of L1Md_T [40] often drives fusion transcripts in the mutant spermatocytes. Our results indicate that transcriptional silencing of retrotransposons is important for the maintenance of not only genomic integrity, but also transcriptomic integrity in meiotic male germ cells.

In conclusion, our results indicate that the combinatorial use of the piRNA system and DNA methylation effectively counteracts retrotransposon activities in developing male germ cells. In prospermatogonia, piRNA-guided *de novo* DNA methylation occurs only in a small subset of retrotransposons, and piRNA-guided RNA cleavage is the major mechanism underlying retrotransposon silencing. In spermatocytes, however, although piRNA-guided RNA cleavage also plays a role, transcriptional silencing by DNA methylation becomes far more important. Thus, there is a shift in the relative contribution of transcriptional and posttranscriptional mechanisms to retrotransposon silencing along with the dynamic epigenome changes during male germ cell development.

## Materials and methods

### Mice

*Pld6* KO and *Dnmt3l* KO mice were described previously [19, 59]. These mutant lines were backcrossed to C57BL/6J for more than 12 generations. To obtain mutant mice of the (MSM/Ms × C57BL/6J) F1 background, we crossed heterozygous mice with MSM/Ms mice for six generations, followed by crossing with heterozygous mice of the C57BL/6J background. The animal experiments were conducted according to Japanese Act on Welfare and Management of Animals, Guidelines for Proper Conduct of Animal Experiments (published by Science Council of Japan), Fundamental Guidelines for Proper Conduct of Animal Experiment and Related Activities in Academic Research Institutions (published by Ministry of Education, Culture, Sports, Science and Technology, Japan), Regulation for Animal Experiments at Kyushu University, and Regulation for Animal Experiments at Nagoya University. The protocols have been approved by Animal Experiments Committee in Kyushu University (A26-010-3) and Nagoya University (2016070501, 2017030268).

### Germ cell preparation

Spermatogonia were isolated from P7 testes by FACS, using a FACSAria II instrument (BD Biosciences), an anti-EpCAM antibody, and a secondary antibody labeled with Alexa Fluor 488, as described previously [27]. The high purity of cell preparations was validated by measuring the DNA methylation level at the *Lit1* differentially methylated region (S12 Fig), which should be unmethylated in male germ cells and 50% methylated in somatic cells. Spermatogonia, spermatocytes, and round spermatids were isolated from P21 testes by FACS, as described previously [60]. The differentiation stages of the collected cells were confirmed by immunostaining with anti-SYCP3 and anti-γ-H2AX antibodies.

### Whole-genome bisulfite shotgun sequencing

Genomic DNA (60–130 ng) was prepared from P7 spermatogonia (about 15,000 cells) using the standard procedure involving proteinase K digestion, phenol/chloroform extraction, and ethanol precipitation. Libraries for whole-genome bisulfite shotgun sequencing were prepared using the post-bisulfite adaptor tagging method [61]. Single-end 100-bp sequencing was performed using HiSeq2500 (Illumina). The software used to generate bcl files included RTA 1.71.21.3 and HCS 2.0.12.0. Approximately 130–150 million reads were obtained for each sample, with an average sequencing depth of about 1.7. After removing the first 6 nt, last 15 nt, and any additional low-quality stretches (Q score < 30), Bismark [62] was used with default parameters for the analysis of methylation in unique regions of the mouse genome (mm10). The mapping efficiency of the datasets was 54.1%–69.9% and the bisulfite conversion rate of the samples was 99.2%–99.3%. Biological replicates for each category ([WT] n = 3, *Pld6*^+/+^, *Pld6*^*+/-*^, and *Dnmt3l*^*+/-*^ mice; [Pld6 KO] n = 2; [Dnmt3l KO] n = 2) showed highly concordant DNA methylation patterns (R = 0.94 to 0.97). To analyze methylation at retrotransposons specific to the genus *Mus*, the consensus sequences listed in the RepeatMasker library were downloaded from RepBase [63]. The retrotransposon sequences and sequencing reads were appropriately processed, and mapping was performed using Bowtie2 [64] with the default parameters. The output files were used to determine the methylation statuses of individual CpG sites. The published whole-genome bisulfite shotgun sequencing data for E13.5 PGCs and E16.5 prospermatogonia [32], *Piwil2* KO spermatogonia [28], and *Pwil4* KO spermatogonia [29] were downloaded from the DDBJ Sequence Read Archive (http://trace.ddbj.nig.ac.jp/dra/index.html).

### polyA(+) RNA-seq, small RNA-seq and 5'-RACE-seq analyses

Total RNA was isolated from P0 testes and FACS-purified P21 germ cells by acid/phenol extraction using Isogen (Nippon Gene).

The libraries for polyA(+) RNA-seq were prepared from 2 μg (P0 testes) or 20 ng (L/Z spermatocytes) of total RNAs (RIN score > 9) using the TruSeq stranded mRNA LT Sample Prep kit (Illumina), and were sequenced using HiSeq1500 in a rapid mode. The sequencing runs were 100-bp paired-end for P0 testes (15–40 million read pairs per sample) and 50-bp single-end for L/Z spermatocytes (9–21 million reads per sample). TopHat2 and Cuffdiff [65, 66] were used for gene expression analysis. The mapping efficiency was 90%–98%. To analyze retrotransposon expression, reads were mapped to a list of retrotransposon consensus sequences by Bowtie2 using the following options: -L 10, -a, -D 20, -R 20, and -i S,1,1.15. For reads that showed the best mapping score with more than one retrotransposon, the read counts were divided by the number of the corresponding retrotransposons, to calculate reads per kilobases per million mapped reads (RPKM). For retrotransposons showing very low expression in the WT control (RPKM^WT^ <1), the fold change was calculated by adding an identical number to both the numerator and denominator of RPKM^KO^/RPKM^WT^, so that the denominator became 1. This alleviated possible experimental fluctuations in fold change calculated using low RPKMs. For P0 testes, we used the first 50-bp sequences of the first read of the 100-bp paired-end reads.

Small RNA-seq libraries were prepared from 400–600 ng of total RNA from P0 testes using NEBNext small RNA kit (New England Biolabs). Small RNA-seq libraries for spermatogonia and L/Z spermatocytes were prepared from 6–42 ng of total RNAs (24,000–150,000 cells) using the TruSeq Small RNA Sample Prep kit (Illumina). These libraries were sequenced using HiSeq1500 in a rapid mode for 50-bp single-end runs (yielding 14–62 million reads per sample). Reads corresponding to abundant noncoding RNA sequences, such as rRNAs and tRNAs, and miRNAs were removed using SeqMap [67], and the remaining reads with a length of 24–33 nt were then mapped to the retrotransposon consensus sequences that were used for the mRNA analysis by SeqMap, allowing two mismatches. For reads showing the least mismatches with more than one retrotransposon, the read counts were divided by the number of the corresponding retrotransposons, to calculate reads per million reads (RPM).

5'-RACE-seq libraries were prepared from 700 ng of total RNA from P0 testes as described previously [35, 36] with some modifications. After removal of rRNAs using Ribo-zero Gold H/M/R kit (Illumina), an RNA adaptor (5'- GUUCAGAGUUCUACAGUCCGACGAUC-3'; SR adaptor) was ligated to RNAs using NEBNext small RNA kit. The ligated RNAs were used as a template for reverse transcription with random primers (5'-AGACGTGTGCTCTTCCGATCTNNNNNN-3'). Resulting cDNAs were amplified by PCR using NEBNext small RNA kit, and DNA fragments of 250–400 bp were purified from an agarose gel. The libraries were sequenced using HiSeq1500 in a rapid mode for 50-bp single-end runs. After removing the adaptor sequence and low quality bases, the reads were mapped to the retrotransposon consensus sequences by bowtie2 and to the mouse genome (mm10) by TopHat2.

### Nascent RNA analysis

Testes collected at P0 were treated with 0.125% trypsin and 0.5 mM EDTA to obtain cell suspensions. After washing with phosphate-buffered saline supplemented with 10% fetal bovine serum, the cells were incubated in DMEM supplemented with 10% fetal bovine serum and 0.5 mM ethynyl uridine for 30 min at 32°C using the Click-iT Nascent RNA Capture Kit (Life Technologies). The treated cells were harvested and RNA was extracted immediately using Isogen. Ethynyl uridine-labeled nascent RNAs were purified according to the manufacturer’s instructions. cDNA was synthesized on beads using the PrimeScript RT reagent Kit with gDNA Eraser (Takara Bio), and analyzed by real-time PCR using primers specific for each retrotransposon (S5 Table).

### Data availability

The deep-sequencing data have been deposited in the Gene Expression Omnibus (GEO) under the accession number GSE70891.

## Supporting information

S1 Fig Global DNA methylation levels in *Pld6* KO and *Dnmt3l* KO germ cells. Distribution of average DNA methylation levels in 100-kb genomic windows for WT PGCs at E13.5 (A), WT prospermatogonia at E16.5 (B), and WT (C), *Pld6* KO (D), and *Dnmt3l* KO (E) spermatogonia at P7. Methylation data for E13.5 and E16.5 were obtained from Kobayashi *et al*. 2013 [32].
(PNG)

S2 Fig DNA methylation profiles in *Pld6* KO and *Dnmt3l* KO germ cells. DNA methylation levels in FACS-purified WT (A), *Pld6* KO (B), and *Dnmt3l* KO (C) spermatogonia at P7 and WT prospermatogonia at E16.5 (D) and their relationship with GC content. (E) DNA methylation profiles along chromosome 1 at the indicated stages. All analyses were performed in 100 kb windows. Methylation data for E13.5 and E16.5 were obtained from Kobayashi *et al*. 2013 [32].
(PNG)

S3 Fig Bisulfite-PCR analysis of retrotransposons in *Pld6* KO and *Dnmt3l* KO spermatogonia. The methylation statuses of selected retrotransposons in P7 spermatogonia are shown (A–H). The PCR primers are listed in S6 Table. The numbers in parentheses indicate methylation levels. Methylated and unmethylated CpG sites are represented by closed and open circles, respectively. Each row represents a single clone.
(PNG)

S4 Fig *De novo* methylation of retrotransposons in E16.5 prospermatogonia and P7 spermatogonia. The extent of *de novo* methylation at individual retrotransposons is compared between WT E16.5 prospermatogonia and *Dnmt3l* KO P7 spermatogonia (orange) and between WT E16.5 prospermatogonia and WT P7 spermatogonia (gray). The methylation levels in E13.5 PGCs were subtracted from those in E16.5 prospermatogonia and P7 spermatogonia. The data for E16.5 prospermatogonia were from Kobayashi *et al*. 2013 [32]. In *Dnmt3l* KO spermatogonia, each retrotransposon shows a methylation level that is very similar to that observed in WT E16.5 prospermatogonia (*R* = 0.81), which is consistent with the findings in unique sequence regions (see Fig 1D).
(PNG)

S5 Fig Comparison of the effects of the *Pld6* KO mutation with those of the *Piwil2* KO and *Piwil4* KO mutations. (A,B) Comparison of the extent of *de novo* methylation at individual retrotransposons between *Pld6* KO and *Piwil2* KO germ cells (A) and between *Pld6* KO and *Piwil4* KO germ cells (B). The data for the *Piwil2* KO and *Piwil4* KO germ cells were from Molaro *et al*. 2014 [28] and Manakov *et al*. 2015 [29]. The dashed line denotes the y = x slope. (C,D) Comparison of fold increases in retrotransposon expression in *Pld6* KO L/Z spermatocytes with those in P10 testes of *Piwil2* KO mutants (C) and *Piwil4* KO mutants (D). The expression data of *Piwil4* and *Piwil2* were from Manakov *et al*. 2015 [29]. Note that P10 testes are composed mainly of somatic cells and spermatogonia, and spermatocytes are a minor population. We also note that the *Piwil4* KO and *Piwil2* KO sequencing reads could not determine the transcribed strand, so the expression levels calculated were the sum of sense and antisense RNAs.
(PNG)

S6 Fig L1 RNA expression analysis for P0 testes by qRT-PCR. Total RNA was reverse transcribed with random primer, and cDNA levels were determined by quantitative PCR for L1Md_A regions in *Pld6* KO (left, blue) and *Dnmt3l* KO (right, orange) testes. The *ActB* mRNA level was used as an internal control. The numbers indicate L1 regions as shown in Fig 2E and 2F.
(PNG)

S7 Fig Expression of individual retrotransposons in double KO testes. Fold changes in the expression of individual retrotransposons are compared between *Pld6* KO and double KO testes (A) and between *Dnmt3l* KO and double KO testes (B). Spots numbered 1–8 are as in Fig 2A. DKO, double KO.
(PNG)

S8 Fig Relationship between retrotransposon derepression in *Pld6* KO and *Dnmt3l* KO testes (P0), piRNA abundance (P0), and decrease in methylation (P7). (A) Length profiles of small RNAs present in P0 testes of WT (black) and *Pld6* KO (light blue) mice. (B) The averages for expression levels of retrotransposon piRNA (24- to 33-nt RNAs) in *Pld6* KO testes relative to those in WT testes. The error bar represents standard deviation. (C) Relationship between the increase in mRNA level and decrease in antisense piRNAs in *Pld6* KO testes at P0. Retrotransposons are grouped according to the extent of the decrease in antisense piRNAs. The box plot features are as described in Fig 4D. The asterisk indicates significant differences between groups (*P* < 0.05, *U* test). NS, not significant. (D) Relationship between the increase in expression in *Dnmt3l* KO newborn testes and the decrease in methylation in *Dnmt3l* KO P7 spermatogonia. Retrotransposons are grouped according to the extent of the decrease in methylation in *Dnmt3l* KO spermatogonia compared with WT spermatogonia.
(PNG)

S9 Fig FACS profiles of postnatal germ cells. Representative FACS profiles are shown for the germ cells from WT (A), *Pld6* KO (B), and *Dnmt3l* KO (C) P21 testes. Cell suspensions were stained with Hoechst-33342 and analyzed as described previously (Gaysinskaya *et al*. 2014 [60]). SG, spermatogonia (green); preL, preleptotene (red); L/Z, leptotene/zygotene (purple); P/D, pachytene/diplotene (green); MII, metaphase II (light blue); RS, round spermatid (blue).
(PNG)

S10 Fig RefSeq gene expression profiles in *Pld6* KO and *Dnmt3l* KO newborn testes. (A) Expression of protein-coding and noncoding genes in Pld6 KO and WT newborn testes. Genes showing increased or decreased expression in *Pld6* KO testes are colored in blue (>2-fold or <1/2, q-value < 0.05). (B) Relationship between the increase in gene expression and the decrease of antisense piRNAs in *Pld6* KO testes at P0. Genes are grouped according to the extent of the decrease in antisense piRNAs. The box plot features are as in Fig 4D. (C) Expression of protein-coding and noncoding genes in *Dnmt3l* KO and WT newborn testes. Genes showing increased or decreased expression in *Dnmt3l* KO testes are colored in red (>2-fold or <1/2, q-value < 0.05). (D) Promoter methylation levels in *Dnmt3l* KO and WT spermatogonia. The red spots indicate the promoters of genes showing increased expression in *Dnmt3l* KO newborn testes (>2-fold, q-value < 0.05).
(PNG)

S11 Fig Allelic expression of RefSeq genes with a strain-specific retrotransposon insertion in *Pld6* KO and *Dnmt3l* KO spermatocytes. Allelic expression was examined by polyA(+) RNA sequencing in P21 L/Z spermatocytes from F1 hybrid mice (MSM/Ms × C57BL/6J) using single-nucleotide polymorphisms. In all genes analyzed, the nearby retrotransposon is absent in the MSM/Ms genome. The determined allelic ratios are shown as pie charts (blue, C57BL/6J; red, MSM/Ms). *ActB* is a control gene without retrotransposon insertion/deletion, showing almost 1:1 allelic ratios in WT, *Pld6* KO, and *Dnmt3l* KO spermatocytes. B6, C57BL/6J; MSM, MSM/Ms.
(PNG)

S12 Fig Bisulfite-PCR analysis of the *Lit1* differentially methylated region. The methylation statuses of the *Lit1* differentially methylated region in WT and *Pld6* and *Dnmt3l* KO spermatogonia are shown. It is known that the region is unmethylated in male germ cells and 50% methylated in somatic cells; thus, its methylation status is used as an indicator of somatic cell contamination in male germ cell preparations. Details are as in S3 Fig. The number of methylated clones and total clones is indicated in parentheses.
(PNG)

S1 Table Methylation, expression, and piRNA abundance of retrotransposons. (XLSX)

S2 Table CpG density, methylation, and expression of RefSeq genes. (XLSX)

S3 Table Genes upregulated by neighboring retrotransposons in *Pld6* KO LZ spermatocytes. (XLSX)

S4 Table Genes upregulated by neighboring retrotransposons in *Dnmt3l* KO LZ spermatocytes. (XLSX)

S5 Table PCR primer sequences used in quantitative PCR analysis shown in Fig 2E, 2F and S6 Fig. (XLSX)

S6 Table PCR primer sequences used in bisulfite-PCR analysis shown in S3 Fig. (XLSX)
