# Supplementary figures and images for: Switching of dominant retrotransposon silencing strategies from posttranscriptional to transcriptional mechanisms during male germ-cell development in mice
Source: PLoS Genet. 2017 Jul 27;13(7):e1006926. doi: 10.1371/journal.pgen.1006926 (PMC5549759; doi:10.1371/journal.pgen.1006926)

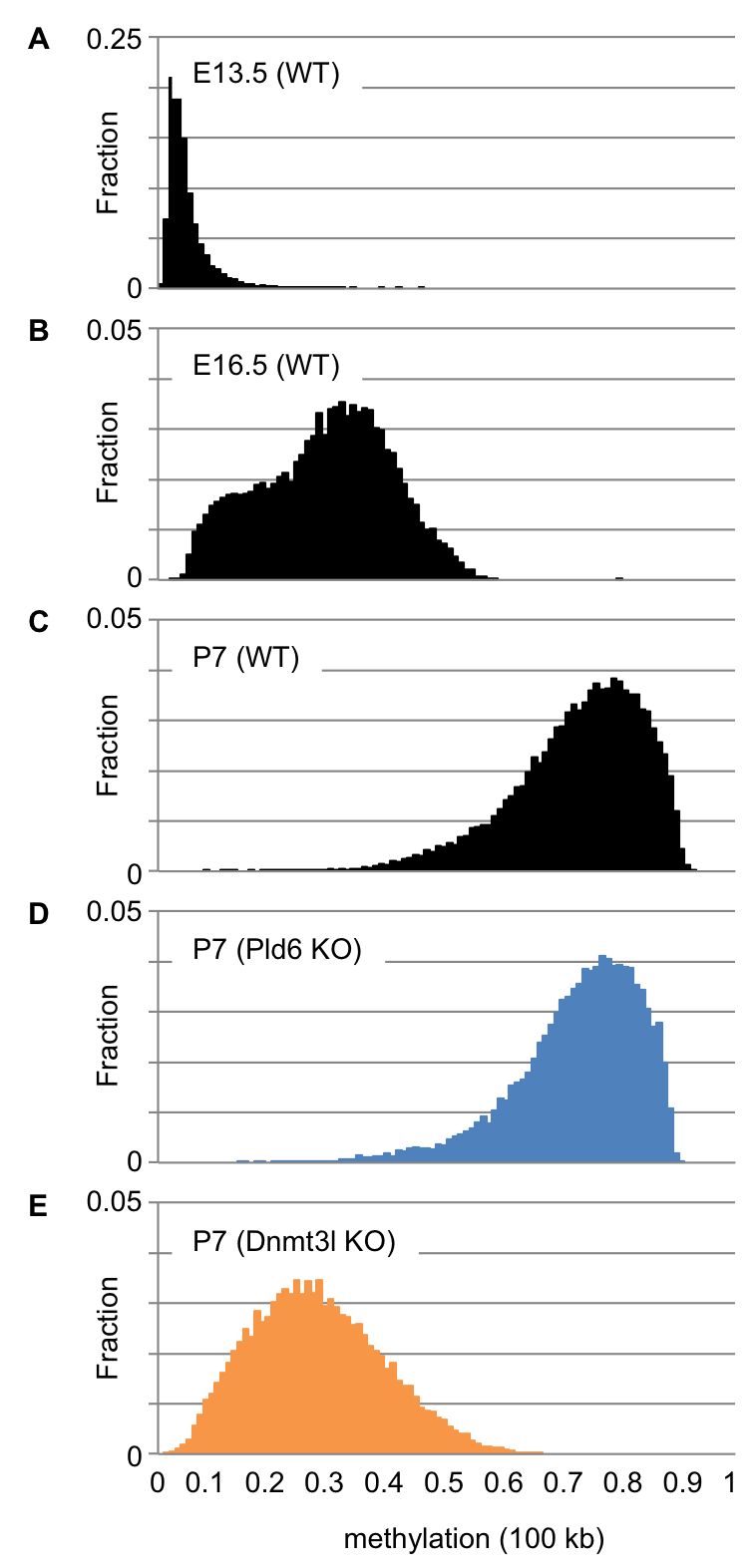

Supplement: S1 Fig — Distribution of average DNA methylation levels in 100-kb genomic windows for WT PGCs at E13.5 (A), WT prospermatogonia at E16.5 (B), and WT (C), Pld6 KO (D), and Dnmt3l KO (E) spermatogonia at P7. Methylation data for E13.5 and E16.5 were obtained from Kobayashi et al. 2013 [32]. (PNG) [file pgen.1006926.s001.png]

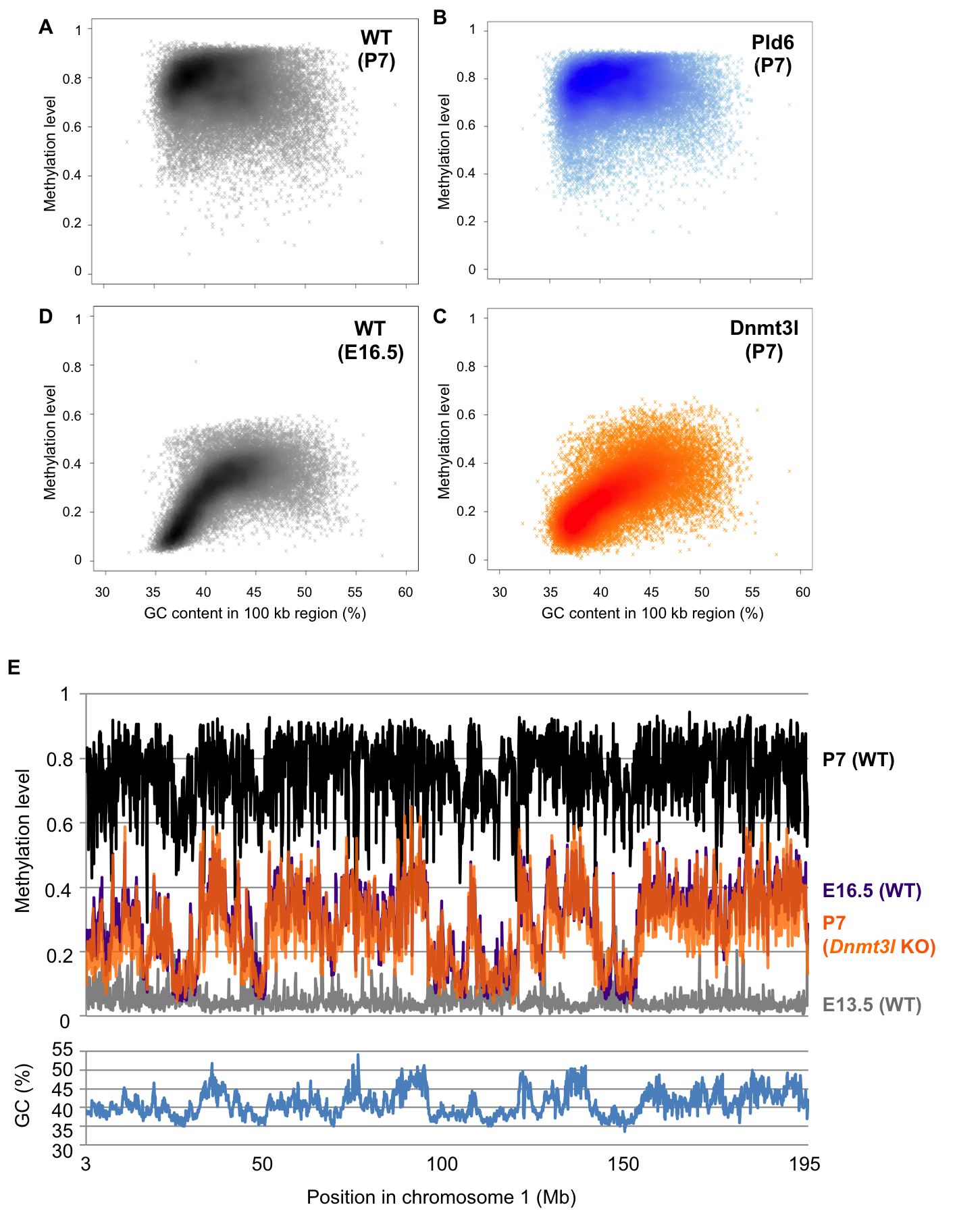

Supplement: S2 Fig — DNA methylation levels in FACS-purified WT (A), Pld6 KO (B), and Dnmt3l KO (C) spermatogonia at P7 and WT prospermatogonia at E16.5 (D) and their relationship with GC content. (E) DNA methylation profiles along chromosome 1 at the indicated stages. All analyses were performed in 100 kb windows. Methylation data for E13.5 and E16.5 were obtained from Kobayashi et al. 2013 [32]. (PNG) [file pgen.1006926.s002.png]

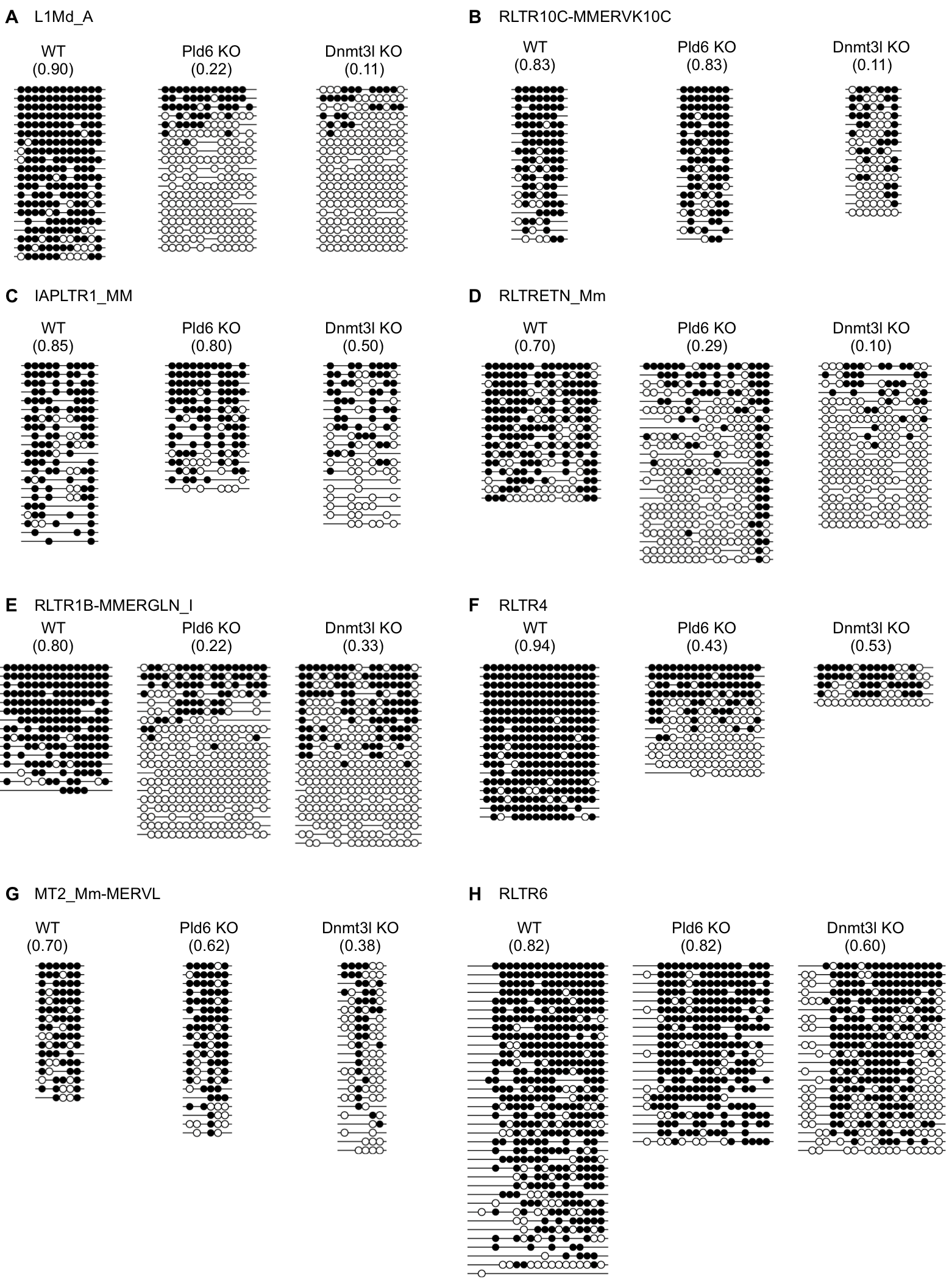

Supplement: S3 Fig — The methylation statuses of selected retrotransposons in P7 spermatogonia are shown (A–H). The PCR primers are listed in S6 Table. The numbers in parentheses indicate methylation levels. Methylated and unmethylated CpG sites are represented by closed and open circles, respectively. Each row represents a single clone. (PNG) [file pgen.1006926.s003.png]

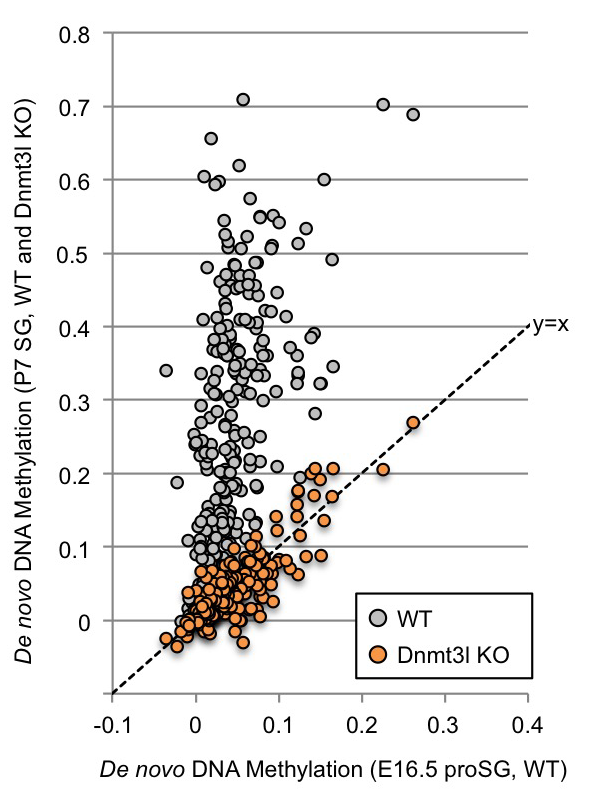

Supplement: S4 Fig — The extent of de novo methylation at individual retrotransposons is compared between WT E16.5 prospermatogonia and Dnmt3l KO P7 spermatogonia (orange) and between WT E16.5 prospermatogonia and WT P7 spermatogonia (gray). The methylation levels in E13.5 PGCs were subtracted from those in E16.5 prospermatogonia and P7 spermatogonia. The data for E16.5 prospermatogonia were from Kobayashi et al. 2013 [32]. In Dnmt3l KO spermatogonia, each retrotransposon shows a methylation level that is very similar to that observed in WT E16.5 prospermatogonia (R = 0.81), which is consistent with the findings in unique sequence regions (see Fig 1D). (PNG) [file pgen.1006926.s004.png]

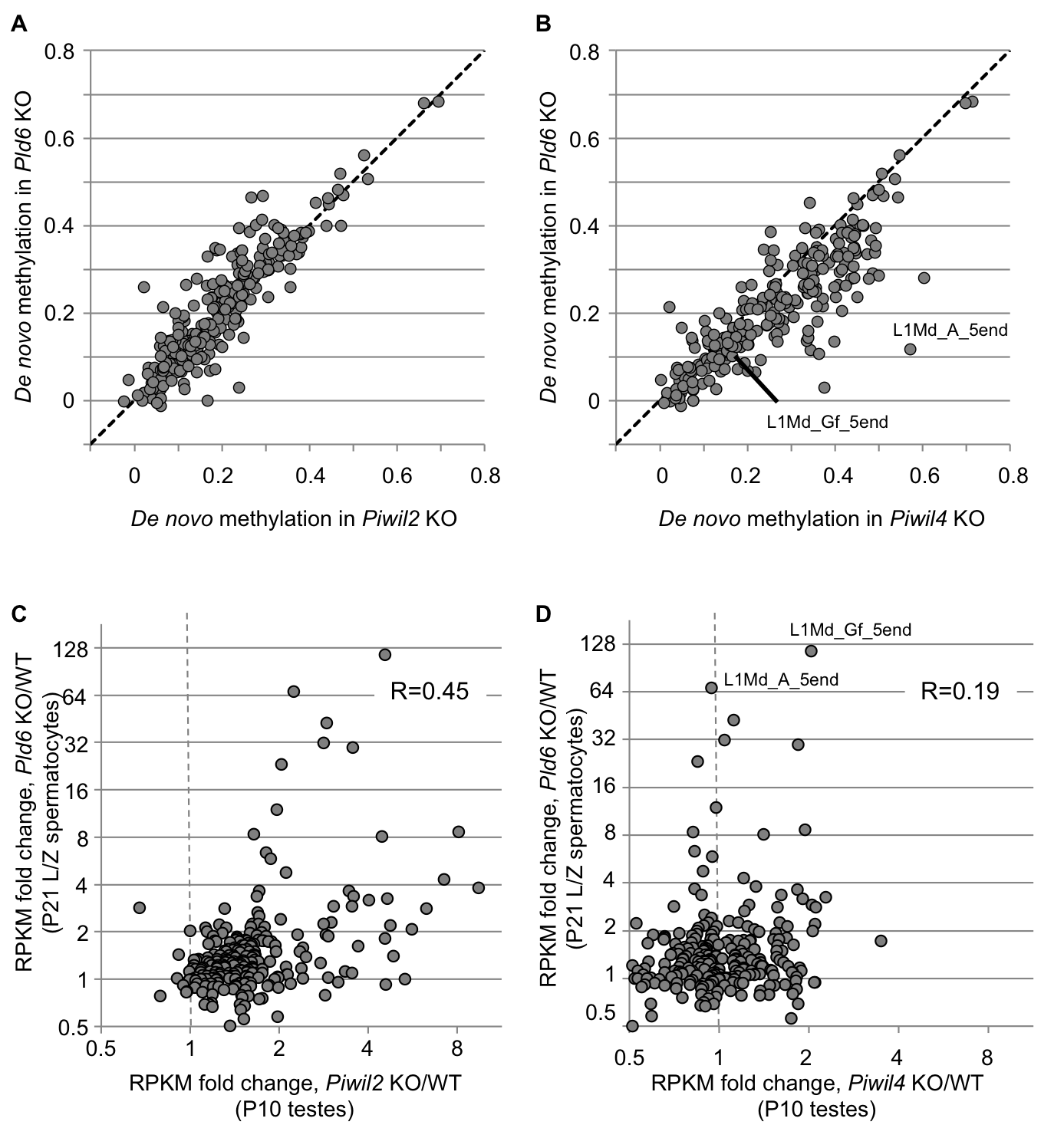

Supplement: S5 Fig — (A,B) Comparison of the extent of de novo methylation at individual retrotransposons between Pld6 KO and Piwil2 KO germ cells (A) and between Pld6 KO and Piwil4 KO germ cells (B). The data for the Piwil2 KO and Piwil4 KO germ cells were from Molaro et al. 2014 [28] and Manakov et al. 2015 [29]. The dashed line denotes the y = x slope. (C,D) Comparison of fold increases in retrotransposon expression in Pld6 KO L/Z spermatocytes with those in P10 testes of Piwil2 KO mutants (C) and Piwil4 KO mutants (D). The expression data of Piwil4 and Piwil2 were from Manakov et al. 2015 [29]. Note that P10 testes are composed mainly of somatic cells and spermatogonia, and spermatocytes are a minor population. We also note that the Piwil4 KO and Piwil2 KO sequencing reads could not determine the transcribed strand, so the expression levels calculated were the sum of sense and antisense RNAs. (PNG) [file pgen.1006926.s005.png]

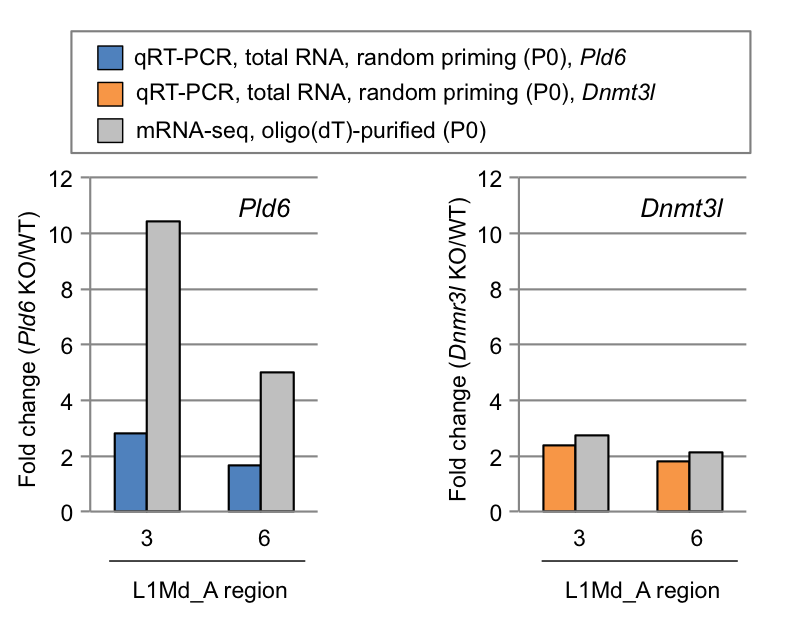

Supplement: S6 Fig — Total RNA was reverse transcribed with random primer, and cDNA levels were determined by quantitative PCR for L1Md_A regions in Pld6 KO (left, blue) and Dnmt3l KO (right, orange) testes. The ActB mRNA level was used as an internal control. The numbers indicate L1 regions as shown in Fig 2E and 2F. (PNG) [file pgen.1006926.s006.png]

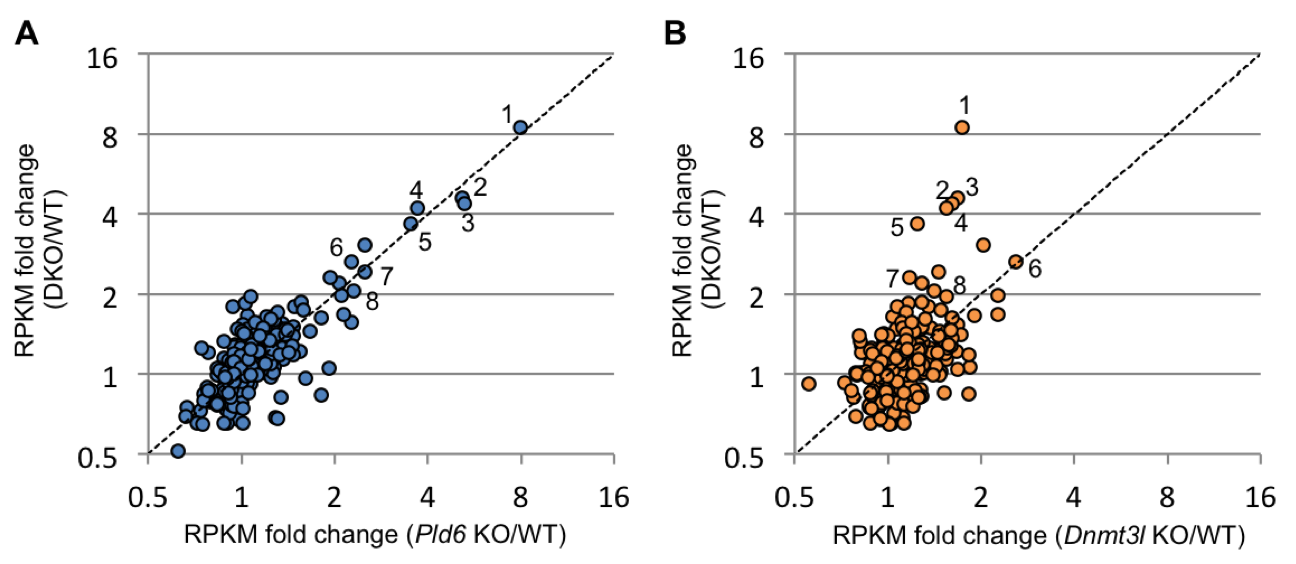

Supplement: S7 Fig — Fold changes in the expression of individual retrotransposons are compared between Pld6 KO and double KO testes (A) and between Dnmt3l KO and double KO testes (B). Spots numbered 1–8 are as in Fig 2A. DKO, double KO. (PNG) [file pgen.1006926.s007.png]

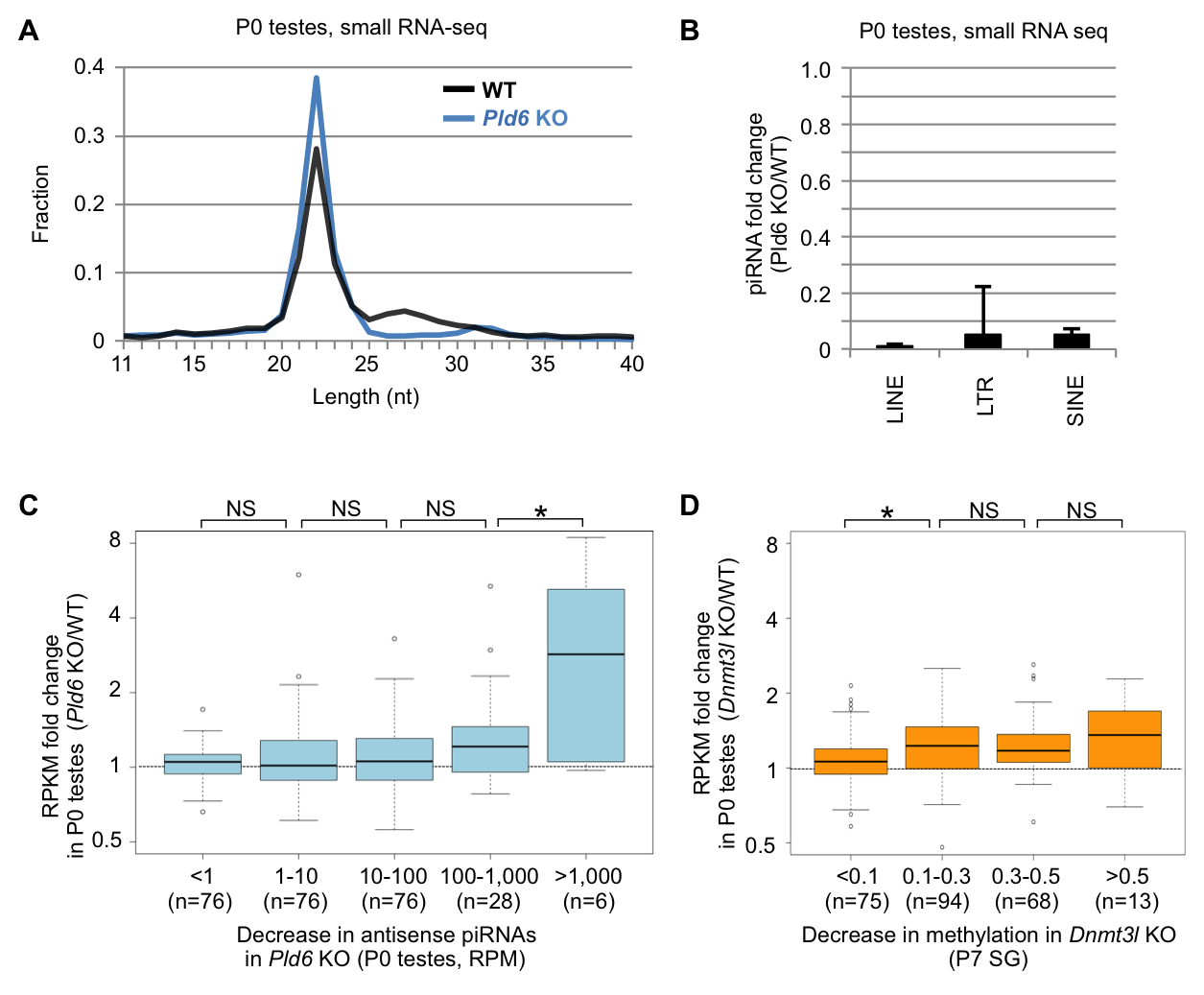

Supplement: S8 Fig — (A) Length profiles of small RNAs present in P0 testes of WT (black) and Pld6 KO (light blue) mice. (B) The averages for expression levels of retrotransposon piRNA (24- to 33-nt RNAs) in Pld6 KO testes relative to those in WT testes. The error bar represents standard deviation. (C) Relationship between the increase in mRNA level and decrease in antisense piRNAs in Pld6 KO testes at P0. Retrotransposons are grouped according to the extent of the decrease in antisense piRNAs. The box plot features are as described in Fig 4D. The asterisk indicates significant differences between groups (P < 0.05, U test). NS, not significant. (D) Relationship between the increase in expression in Dnmt3l KO newborn testes and the decrease in methylation in Dnmt3l KO P7 spermatogonia. Retrotransposons are grouped according to the extent of the decrease in methylation in Dnmt3l KO spermatogonia compared with WT spermatogonia. (PNG) [file pgen.1006926.s008.png]

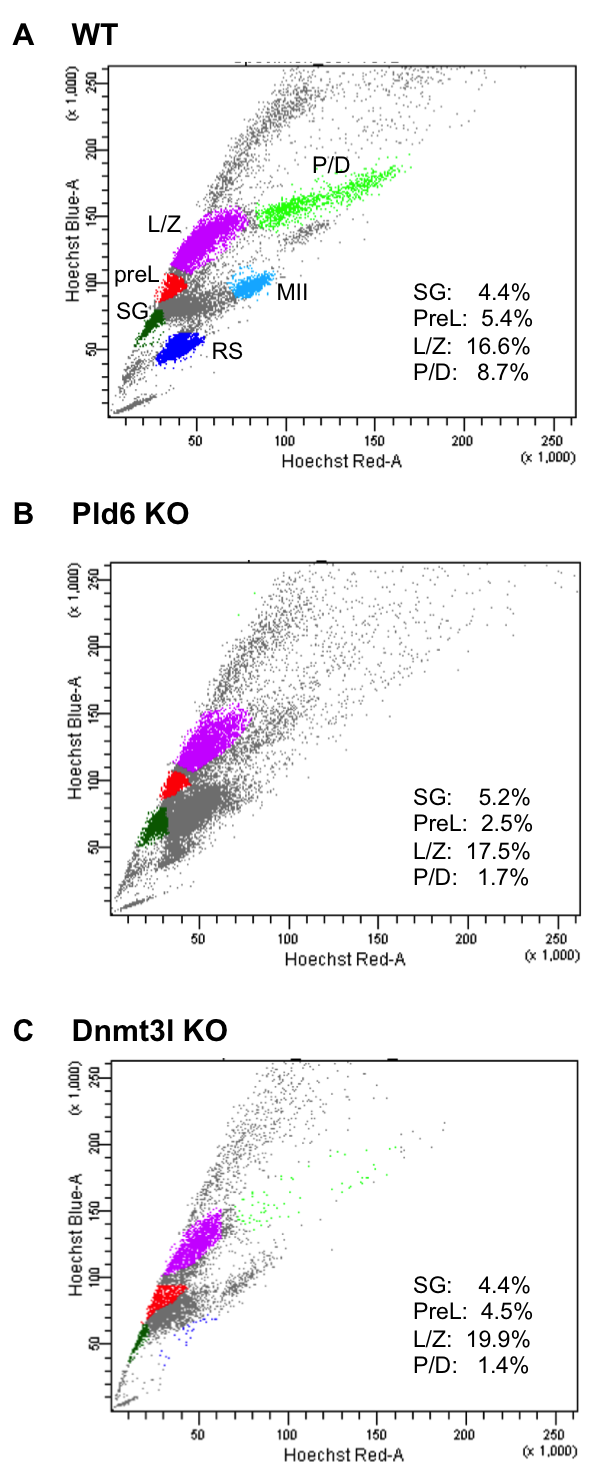

Supplement: S9 Fig — Representative FACS profiles are shown for the germ cells from WT (A), Pld6 KO (B), and Dnmt3l KO (C) P21 testes. Cell suspensions were stained with Hoechst-33342 and analyzed as described previously (Gaysinskaya et al. 2014 [60]). SG, spermatogonia (green); preL, preleptotene (red); L/Z, leptotene/zygotene (purple); P/D, pachytene/diplotene (green); MII, metaphase II (light blue); RS, round spermatid (blue). (PNG) [file pgen.1006926.s009.png]

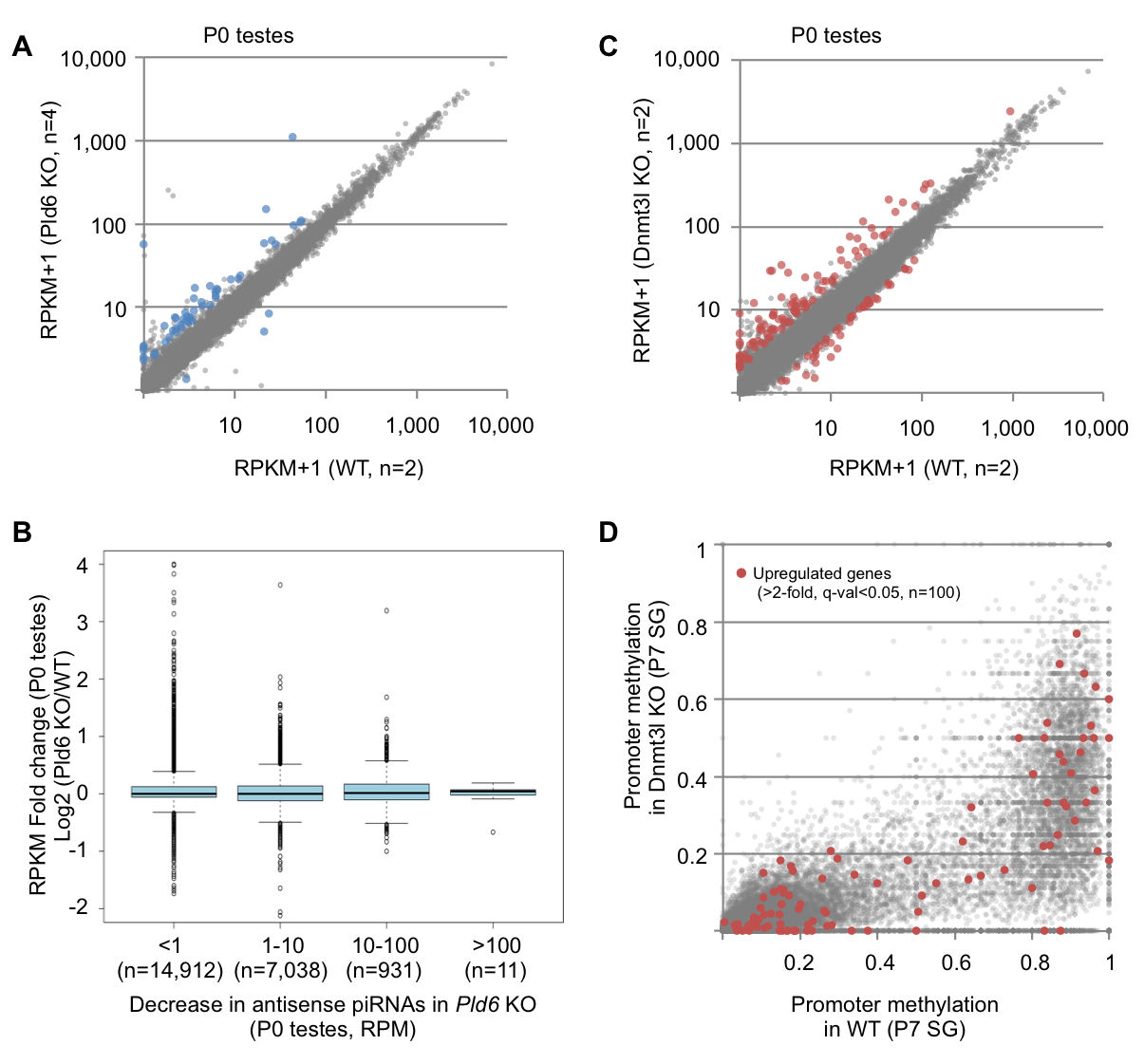

Supplement: S10 Fig — (A) Expression of protein-coding and noncoding genes in Pld6 KO and WT newborn testes. Genes showing increased or decreased expression in Pld6 KO testes are colored in blue (>2-fold or <1/2, q-value < 0.05). (B) Relationship between the increase in gene expression and the decrease of antisense piRNAs in Pld6 KO testes at P0. Genes are grouped according to the extent of the decrease in antisense piRNAs. The box plot features are as in Fig 4D. (C) Expression of protein-coding and noncoding genes in Dnmt3l KO and WT newborn testes. Genes showing increased or decreased expression in Dnmt3l KO testes are colored in red (>2-fold or <1/2, q-value < 0.05). (D) Promoter methylation levels in Dnmt3l KO and WT spermatogonia. The red spots indicate the promoters of genes showing increased expression in Dnmt3l KO newborn testes (>2-fold, q-value < 0.05). (PNG) [file pgen.1006926.s010.png]

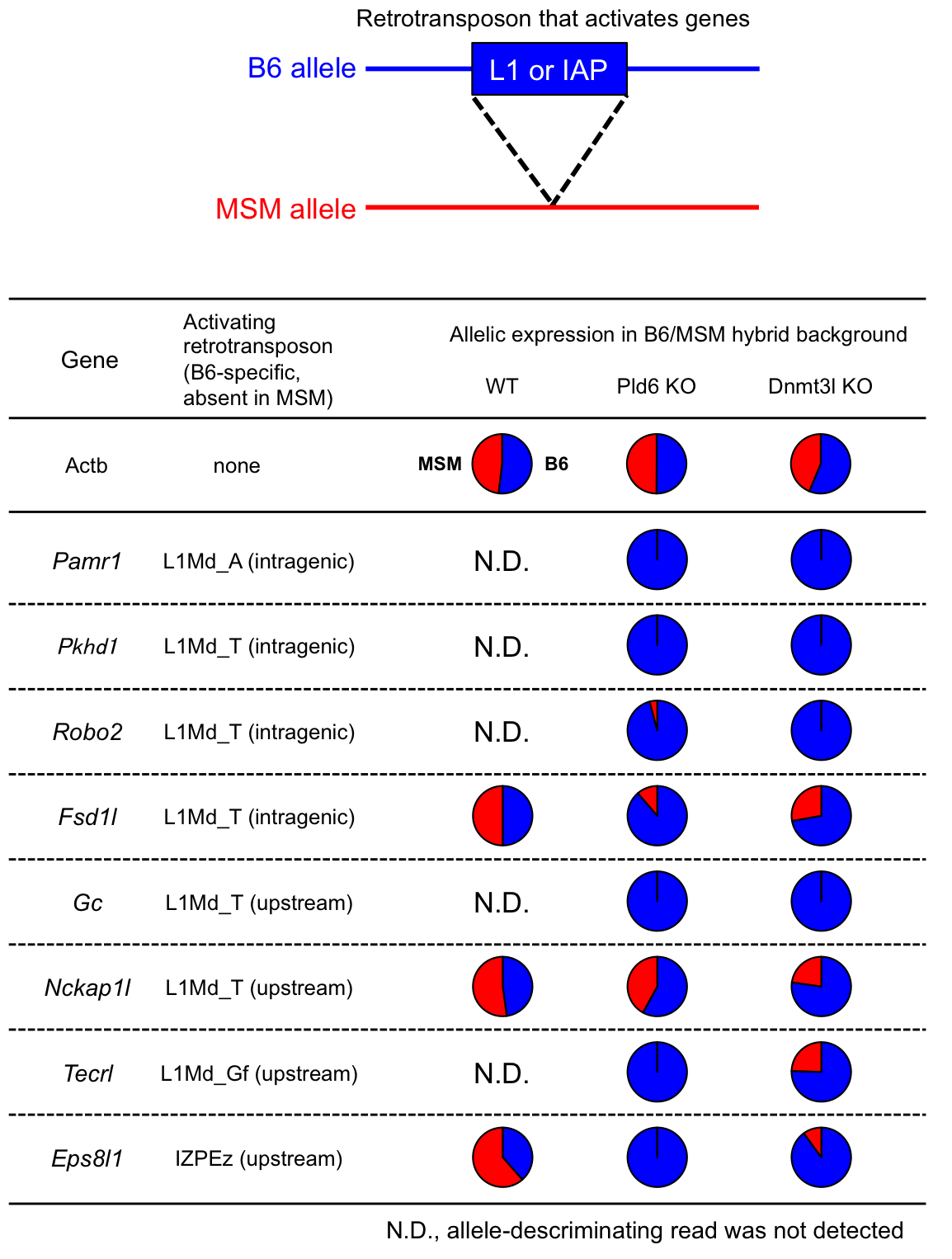

Supplement: S11 Fig — Allelic expression was examined by polyA(+) RNA sequencing in P21 L/Z spermatocytes from F1 hybrid mice (MSM/Ms × C57BL/6J) using single-nucleotide polymorphisms. In all genes analyzed, the nearby retrotransposon is absent in the MSM/Ms genome. The determined allelic ratios are shown as pie charts (blue, C57BL/6J; red, MSM/Ms). ActB is a control gene without retrotransposon insertion/deletion, showing almost 1:1 allelic ratios in WT, Pld6 KO, and Dnmt3l KO spermatocytes. B6, C57BL/6J; MSM, MSM/Ms. (PNG) [file pgen.1006926.s011.png]

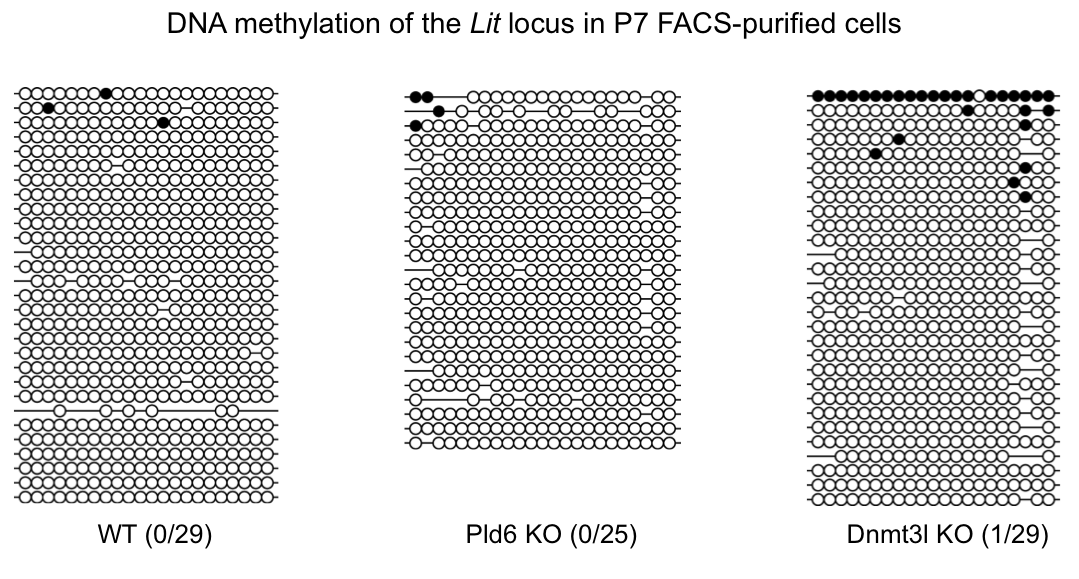

Supplement: S12 Fig — The methylation statuses of the Lit1 differentially methylated region in WT and Pld6 and Dnmt3l KO spermatogonia are shown. It is known that the region is unmethylated in male germ cells and 50% methylated in somatic cells; thus, its methylation status is used as an indicator of somatic cell contamination in male germ cell preparations. Details are as in S3 Fig. The number of methylated clones and total clones is indicated in parentheses. (PNG) [file pgen.1006926.s012.png]
